# Looking both ways: a review of methods for assessing research impacts on policy and the policy utilisation of research

**DOI:** 10.1186/s12961-018-0310-4

**Published:** 2018-06-25

**Authors:** Robyn Newson, Lesley King, Lucie Rychetnik, Andrew Milat, Adrian Bauman

**Affiliations:** 1 Sydney School of Public Health, The University of Sydney, Charles Perkins Centre D17, Level 6 Hub, Sydney, NSW 2006 Australia; 2 School of Medicine Sydney, University of Notre Dame Australia, 160 Oxford St, Darlinghurst, 2010 Australia

**Keywords:** Research impact assessment, Research impact, Research payback, Policy impact, Research utilisation, Research use, Health policy, Health research, Evidence-informed policy

## Abstract

**Background:**

Measuring the policy and practice impacts of research is becoming increasingly important. Policy impacts can be measured from two directions – tracing forward from research and tracing backwards from a policy outcome. In this review, we compare these approaches and document the characteristics of studies assessing research impacts on policy and the policy utilisation of research.

**Methods:**

Keyword searches of electronic databases were conducted in December 2016. Included studies were published between 1995 and 2016 in English and reported methods and findings of studies measuring policy impacts of specified health research, or research use in relation to a specified health policy outcome, and reviews reporting methods of research impact assessment. Using an iterative data extraction process, we developed a framework to define the key elements of empirical studies (assessment reason, assessment direction, assessment starting point, unit of analysis, assessment methods, assessment endpoint and outcomes assessed) and then documented the characteristics of included empirical studies according to this framework.

**Results:**

We identified 144 empirical studies and 19 literature reviews. Empirical studies were derived from two parallel streams of research of equal size, which we termed ‘research impact assessments’ and ‘research use assessments’. Both streams provided insights about the influence of research on policy and utilised similar assessment methods, but approached measurement from opposite directions. Research impact assessments predominantly utilised forward tracing approaches while the converse was true for research use assessments. Within each stream, assessments focussed on narrow or broader research/policy units of analysis as the starting point for assessment, each with associated strengths and limitations. The two streams differed in terms of their relative focus on the contributions made by specific research (research impact assessments) versus research more generally (research use assessments) and the emphasis placed on research and the activities of researchers in comparison to other factors and actors as influencers of change.

**Conclusions:**

The Framework presented in this paper provides a mechanism for comparing studies within this broad field of research enquiry. Forward and backward tracing approaches, and their different ways of ‘looking’, tell a different story of research-based policy change. Combining approaches may provide the best way forward in terms of linking outcomes to specific research, as well as providing a realistic picture of research influence.

**Electronic supplementary material:**

The online version of this article (10.1186/s12961-018-0310-4) contains supplementary material, which is available to authorized users.

## Background

Research evidence has the potential to improve health policy and programme effectiveness, help build more efficient health services and ultimately achieve better population health outcomes [1]. The translation of research evidence into health policy, programmes and services is an ongoing and commonly reported challenge [2]. If research is not translated, it means that extensive investments in research and development are potentially going to waste [3]. In response to this issue, researchers and funding bodies are being asked to demonstrate that funded research represents value for money, not only through the generation of new knowledge but also by contributing to health and economic outcomes [4, 5]. Pressures for accountability have also led to a greater focus on evidence-informed policy-making, which calls for policy-makers to make greater use of research in policy decisions so that policies and programmes are more likely to improve population health outcomes [1].

Consequently, there has been an increasing emphasis on measuring the wider impacts of research [6] (“*an effect on, change or benefit to the economy, society, culture, public policy or services, health, the environment or quality of life, beyond academia*” ([7] p. 4), as well as understanding how research is used in decision-making processes [1, 8–13]. This literature review focuses on methods for measuring the impacts of research on public policy specifically, where policy impacts are considered as intermediary outcomes between research outputs and longer-term impacts such as population health and socioeconomic changes [1]. Health policy impacts can be defined variously, but encompass indirect or direct contributions of research processes or outputs to the development of new health policy or revisions of existing health policy at various levels of governance [14]. It is proposed that the use of research to inform public policy leads to desired outcomes such as health gains [1]. Policy impacts, however, can be more easily measured and attributed to research than impacts that are further ‘upstream’ from research outputs [1, 15].

Measuring the policy impacts of research can be approached from two directions – tracing forward from research to identify its impacts on policy and other outcomes, and tracing backwards from a policy outcome (e.g. policy change or document) to identify whether and how research has been utilised [1, 11, 16, 17]. Several reviews have considered conceptual approaches and methods for assessing research impacts [5, 6, 16–22] and research utilisation in health policy-making [1, 11]. These reviews identify elements that characterise and differentiate assessment processes (Box 1). Examples of the empirical application of forward tracing research impact assessments are more commonly discussed in existing reviews than backward tracing approaches.

In addition, existing reviews have only addressed the relative advantages and disadvantages of forward and backward tracing approaches to a limited degree [1, 11, 16, 17]. Forward tracing approaches are reported to be more common because they allow a more precise focus on specific research, which is important for funding bodies seeking to account for research expenditure [1, 16, 17]. However, this focus on specific research creates challenges attributing any observed changes to the specific research under study [16], this is because research is usually only one factor amongst many at play during policy decisions [25]. Furthermore, where research is influential, policy decisions are usually based on the synthesis of a broad spectrum of knowledge, rather than the findings of individual studies or a specific programme of work [26]. In addition, it can be difficult to establish what would have occurred in the absence of the research under study (counterfactual conditional) [27]; there is no ‘control’ state against which to compare outcomes [18]. Examining the context in which policy change occurs therefore becomes important [27, 28]; however, forward tracing assessments have been criticised for failing to address the complexities involved in policy decision-making [17]. Forward tracing assessments are also subject to limitations associated with the timing of assessment because research impacts can take a long time to occur [25]. On the other hand, backward tracing approaches are said to be more suited to understanding the extent and processes through which knowledge, including research, influences policy decisions [11], but they are not always able to identify the influences of specific research, or the relative degree of influence of a particular study, and other potential limitations in terms of measuring research use are not well documented [23, 24, 29].

**Fig. 1:**
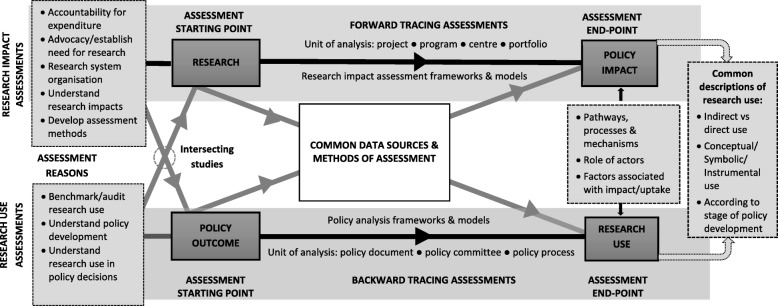
Descriptive framework for research impact and research use assessments

In this review of the literature, our aim was to document the extent and nature of studies measuring the impacts of health research on policy and compare forward and backward tracing approaches to assessment. Firstly, we documented the characteristics of empirical studies drawn from two streams of empirical research, namely studies measuring the impacts of health research on policy and studies examining research utilisation in health policy decisions. Secondly, a descriptive framework (Fig. 1) was developed to allow structured comparisons between assessments to be made. This framework incorporated both the key elements identified in studies described in previous reviews (Box 1) and those emerging from an iterative analysis of studies included in the current review. Thirdly, based on reported strengths and limitations of the approaches described, we considered what may be gained or lost where different approaches were chosen, and particularly how the direction of assessment may influence the assessment findings. Finally, we sought to identify gaps in the existing literature and areas that warranted further investigation. To our knowledge, this paper is the first to systematically analyse these two streams of research in relation to each other.

## Methods

This review of the literature was completed in December 2016, and examines peer-reviewed empirical studies published between 1995 and 2016 in English that measured the impacts of health research on policy and research use in health policy decisions. We also examined existing reviews on these topics. Our review questions were as follows:

- What are the core elements of empirical research impact or research use assessments?
- What is the extent and nature of empirical peer-reviewed research in this area of study?
- What are the advantages and disadvantages of different approaches to assessment?
- Where do the gaps in the existing literature lie and which areas warrant further investigation?

### Search strategy

The review utilised an iterative process that included several steps. We initially searched electronic databases (Medline, CINAHL, EBM reviews, Embase, Google Scholar) using keyword search terms derived from research impact assessment reviews and empirical studies known to the authors (e.g. research impact, impact assessment, investment return, research payback, payback model, payback framework, societal impact, policy impact, research benefit, health research). Based on the abstracts from this search, we compiled all empirical studies that reported policy impacts in relation to health research, or research use in relation to health policy outcomes, and reviews reporting methods of research impact assessment.

After completing the above process, it was clear that the initial search had identified papers starting with research and measuring its impacts, but had been less successful in identifying papers starting with policy outcomes and measuring research use. Another search of key databases was therefore conducted using ‘research use’ search terms derived from the studies already identified on this topic (e.g. research use, research translation, evidence use, research utilisation, research evidence, evidence-based policy, knowledge utilisation, health policy). This resulted in further relevant studies being added to our list.

**Fig. 2:**
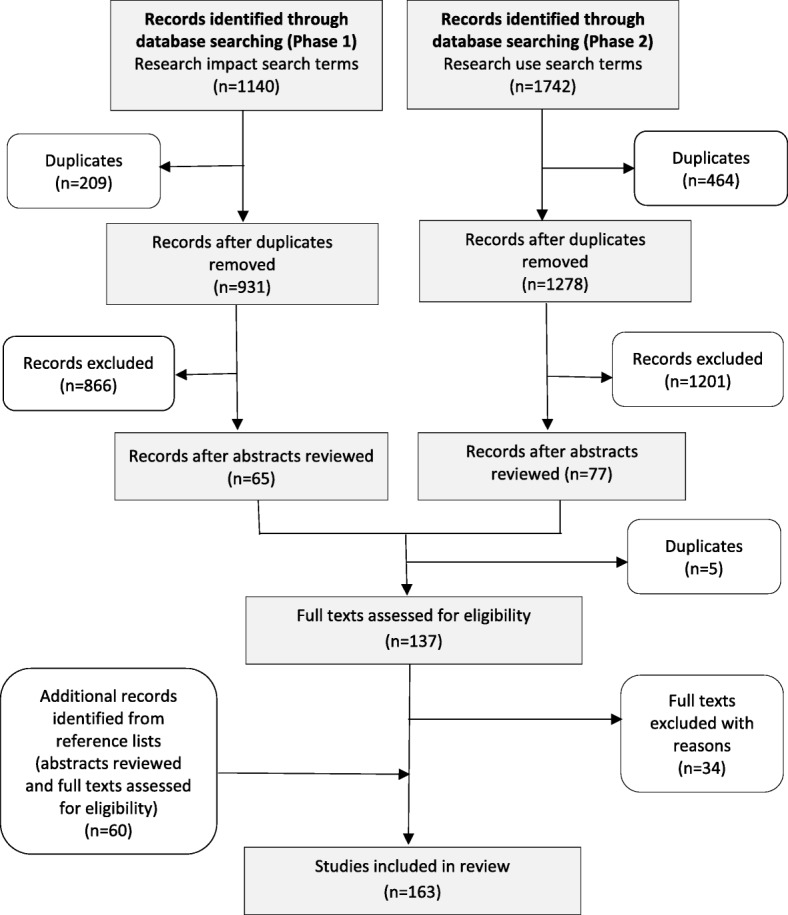
Flow diagram of literature search process

The reference lists of all included studies were then scanned to identify other relevant papers not found during the database search. The full texts of included studies were read to ensure they met the inclusion/exclusion criteria for the review. The search process is shown in Fig. 2.

### Inclusion criteria

In relation to our analysis of empirical studies, we only included studies where the research or health policy outcome under study was clearly defined. We excluded studies that did not report on health research or a health policy outcome. Studies that did not report methods in conjunction with results of impact or research use assessments were also excluded. In addition, we excluded studies reporting opinions about research impact or use in general, rather than measuring the impact of specific research or research use in relation to specific policy outcomes. Finally, we excluded studies examining strategies or interventions to improve research translation. As our aim was to define and report key characteristics of studies rather than synthesise study findings, studies were not included/excluded based on study quality.

### Data extraction, development of the descriptive framework and categorisation of empirical studies

To analyse the included studies, we prepared a data extraction tool incorporating the key elements described in existing reviews (Box 1). The initial categories were progressively refined during the data extraction and analysis process and integrated into a comprehensive ‘research impact and research use’ assessment framework; the elements of which are described in the results below. Thus, data extraction was iterative, until information from all the empirical studies was documented in relation to the final framework. Categorisation of studies according to key elements of the framework was done based on statements made by the study authors, where possible. Where judgements were required, categorisations were discussed by the authors of this paper until a consensus was reached.

## Results

### Literature search

An initial review of abstracts in electronic databases against the inclusion criteria yielded 137 papers, 34 of which were excluded after full text review. Searches of reference lists of the included papers identified a further 60 studies (Fig. 2). The final number of papers included in this review was 163; 144 were empirical studies reporting methods and findings of research use or research impact assessments (included in the results that follow) and 19 were reviews of the literature. A full list of the included empirical studies is provided in Additional file 1. To aid the reader to identify studies cited as examples in the results section, the numbers given in brackets in subscript match the reference numbers in Additional file 1.

### Analysis of empirical studies (*n* = 144)

#### Overview of the descriptive framework and included studies

Figure 1 provides a descriptive representation of the empirical studies included in this review. It depicts the two parallel streams of research, namely studies concerned with measuring and understanding the ‘impacts of research’ (research impact assessments) and those concerned with measuring and understanding ‘research use’ in policy decisions (research use assessments). The study starting point defined whether a study was categorised as research impact or research use – research impact assessments usually started with research and traced forward to identify the benefits arising from that research; conversely, research use assessments usually started with a policy outcome and traced backwards to understand whether and how research had been used. There was a small group of ‘intersecting studies’ that drew on elements from both streams of research, and where, occasionally, research impact assessments used backward tracing approaches and research use assessments used forward tracing approaches. Assessments in both streams were based on similar theoretical concepts, utilised similar methods and had similar assessment end-points (i.e. they reported on similar outcomes). However, outcomes were reported from different perspectives depending on the direction of assessment chosen. The unit of analysis utilised in assessments varied across studies overall, ranging from a narrow focus on specific research projects or policy outputs to a broader focus on larger programmes of research or policy processes.

Below, we describe the number and nature of the included studies according to the key elements of the framework. Table 1 provides the number of studies categorised by type of assessment, direction of assessment, unit of analysis and methods of assessment. Illustrative examples of the different types of assessments are provided in Table 2. Overall, we identified a similar number of research impact (*n* = 68; Table 1) and research use assessments (*n* = 67; Table 1), as well as a small group of intersecting studies, drawing on elements of both streams of research (*n* = 9; Table 1).

**Table 1:** Descriptive characteristics of included studies (*n* = 144)

	Research impact assessments (*n* = 68)	Intersecting studies	Research use assessments (*n* = 67)
		(*n* = 9)	
Direction of assessment			
Forward tracing assessments	61		4
Backward tracing assessments	7		63
Elements of both		9	
Unit of analysis^a^			
Research project	48		4
Research programme	4		–
Research centre	3		–
Research portfolio	4		–
Policy document	6		10
Policy committee	–		4
Policy process	–		49
More than one unit of analysis	3	9	
Methods of assessment			
Framework used to structure assessment			
*Yes*	46	7	23
*No*	22	2	44
Data sources			
*Multiple data sources*	48		46
*Interviews only*	1		5
*Survey only*	13		3
*Desk analysis only*	6		13
Utilised case studies	42	8	53
*Single case study*	8	4	32
*Multiple cases studies*	34	4	21
Respondents^b^			
*Researchers only*	26	1	1
*Decision-makers only*	5		17
*Researchers and decision-makers*	27	5	8
*Broad range of policy actors*	–	3	28

^a^Primary unit of analysis (i.e. findings reported in relation to this unit of analysis)

^b^Not all studies have respondents (e.g. desk-based analyses)

**Table 2:** Illustrative examples of forward and backward tracing assessments, and assessments utilising both approaches

Main assessment reason	Assessment start-point	Conceptual framework and methods	Assessment end-point/outcomes reported	Comment
FORWARD TRACING ASSESSMENTS				
*A: Wooding et al. 2014* ^*[140]*^ *(Australia, Canada, UK)*				
Understand impacts	Research projects from cardiovascular and stroke research funders	Payback Framework (multiple impact categories) 29 randomly selected case studies Data sources: researcher surveys; interviews with researchers and end-users; and external peer review Scoring of impacts for each payback category by an expert panel Qualitative and quantitative analysis of factors associated with impact	Sum of impacts across impact categories, impact scores, plus some specific examples reported Analysis of impact pathways with reference to existing theories and conceptual perspectives Factors explaining variations in impact	Forward tracing, research impact assessment where single projects were the unit of analysis
*B: Kok et al. 2016* ^*[69]*^ *(Netherlands/Ghana)*				
Understand impacts	Research projects that were part of a Ghanaian-Dutch research programme	Contribution Mapping Framework (policy and practice impacts only) 30 case studies (selected in order of funding allocation) Data sources: research proposals, mid-terms reviews and reports; interviews with researchers and end-users	Number of ‘used’ studies Description of how produced knowledge was used Description of research and translation processes associated with the use of produced knowledge	Forward tracing, research impact assessment where single projects were the unit of analysis
*C: Hanney et al. 2013* ^[55]^ *(United Kingdom)*				
Accountability/advocacy Inform research funding strategies	Grants funded by Asthma UK (project grants; professional chairs; fellowship grants; collaborative research centre)	Payback Framework: (multiple impact categories) Survey of 163 researchers; 14 purposely selected case studies Data sources: Researcher survey and interviews; archival and document review; bibliometric analysis	Sum of impacts by impact category and some specific examples reported Comparison of impacts reported by funding mode	Forward tracing, research impact assessment where more than one type of research grant was the unit of analysis Analysis of multiple funding modes and comparison of outcomes
*D: Hanney et al. 2006* ^[53]^ *(United Kingdom)*				
Test methods Understand research impacts	Body of diabetes research published in the early 1980s by one team leader of acknowledged influence	No framework used – broad description of multiple types of impacts Single case study Data sources: bibliometric analysis; surveys and interviews with researchers; critical publication pathway analysis	Description of benefits identified Factors associated with significant impact Methodological issues	Forward tracing, research impact assessment where a programme of research is the unit of analysis
*E: Hanney et al. 2000* ^[51]^ *(United Kingdom)*				
Test methods and model Understand research impacts	Research and development centres funded by a regional office of the National Health Service	Payback Framework (multiple impact categories) 2 purposefully selected case studies Data sources: document analysis; bibliometric analysis; interviews with researchers and end-users	Description of impacts identified Methodological issues	Forward tracing, research impact assessment where research centres were the unit of analysis Used a triangulation approach, combining analysis of selected projects with the broader longer-term contribution of the centre as a whole
*F: Orians et al. 2009* ^*[98]*^ *(United States of America)*				
Test methods and model Accountability	National Institute of Environmental Health Sciences (NIEHS) Division of Extramural Research asthma-related research portfolio	Logic model of pathways linking research to ultimate outcomes (multiple categories of impact) Survey of 725 researchers; interviews with 16 end-users	Sum of impacts reported by impact category and some specific examples reported Methodological issues	Forward tracing, research impact assessment where a portfolio of research was the unit of analysis Combined analysis of the work of researchers who had ever received NEIHS asthma research funding (over a 30-year period) with a broader analysis of awareness and use of any research from the portfolio by end-users
*G: Dobbins et al. 2004* ^[23]^ *(Canada)*				
Understand research use Identify factors associated with use	Systematic reviews disseminated to public health decision-makers through the Effective Public Health Practice Project (EPHPP)	No framework used - policy impacts only Survey of policy-makers who were members of technical review groups Statistical analysis of factors associated with use	Extent of systematic review use and perceived influence on recommendations Factors explaining variations in review use.	Forward tracing, research use assessment where a group of related projects (systematic reviews) were the unit of analysis Systematic reviews commissioned by policy agency to address priority policy questions
BACKWARD TRACING ASSESSMENTS				
*H: Grant et al. 2000* ^[41]^ *(United Kingdom)*				
Test assessment method Understand research impacts	Clinical guidelines on disease management developed in the UK	No framework used Bibliometric analysis of publications cited in 15 guidelines	Number of papers cited and type of papers cited Research characteristics associated with citation	Backward tracing, research impact assessment where policy documents were the unit of analysis
*I: Kite et al. 2014* ^*[68]*^ *(United States of America)*				
Benchmark research use	Documents and oral testimony associated with legislative bills relevant to active living archived by the Minnesota State Legislature	No framework used Content analysis of policy documents	Number of documents mentioning research and other types of information	Backward tracing, research use assessment where policy documents were the unit of analysis
*J: Dakin et al. 2016* ^[17]^ *(United Kingdom)*				
Understand policy decisions	National Institute for Heath and Clinical Excellence (NICE) guidance documents, Health Technology Assessment (HTA) reports and appeal decision reports	Content analysis of 73 NICE appraisals. Statistical analysis estimating the impact of key coded variables on decision-making	Factors associated with decision-making including availability and quality of research	Backward tracing research use assessment where policy documents were the unit of analysis
*K: PausJenssen et al. 2003* ^*[103]*^ *(Canada)*				
Understand policy decisions	Decision-making process of the Drug Quality and Therapies Committee (DQTC) of Ontario	No framework used Single case study – committee meetings between Dec 1997 and Aug 1998 Data sources: interviews with committee members; observation of committee meetings Qualitative analysis of factors associated with decision-making	Factors associated with decision-making including role of research	Backward tracing research use assessment where a committee was the unit of analysis
*L: Williams et al. 2008* ^*[137]*^ *(United Kingdom)*				
Understand research use in policy decisions	Technology appraisal decisions made by the NICE Technology Appraisal Committee and resource allocation decisions concerning adoption of drugs and other therapies made by four local national health service committees	5 case studies of committees: 4 local and one national organisation Data sources: Documentary analysis; observation of committee meetings; committee member workshop discussions and interviews Prospective data collection	Description of extent and nature of use of economic analyses in decision-making Factors associated with the use of research evidence relating to economic analyses	Backward tracing research use assessment where committees were the unit of analysis Compared decision-making at a national and local level
*M: Shearer et al. 2014* ^*[118]*^ *(Burkino Faso)*				
Understand research use in policy decisions	Community integrated management of childhood illness; home management of malaria; removal of user fees for antiretroviral treatment for HIV	No framework used 3 policy case studies Data sources: Surveys with policy actors Network analysis. Statistical analysis of probabilities of research provision and request between actors and actors use of research to inform policy	Conditions under which research is provided and requested Factors associated with research use in policy-making	Backward tracing research use assessment where policy processes were the unit of analysis
*N: Nabyonga-Orem et al. 2014* ^*[91]*^ *(Uganda)*				
Understand research use in policy decisions	Change in malaria drug treatment policy and its implementation in Uganda	No framework used Single case study Data sources: interviews with policy actors and document review Respondents rated degree of consistency between the policy decision and the available evidence	Description of the use of research and other information in the policy decision and by different actors Type and quantity of research cited in policy documents Factors facilitating the uptake of research	Backward tracing research use assessment where a policy process was the unit of analysis
*O: Hyde et al. 2015* ^[61]^ *(United States of America)*				
Understand research use in policy decisions	Development of state-level policies to ensure that youth in foster care receive safe and appropriate psychopharmacological treatment	Used an evidence framework for understanding the different types, applicability and uses of evidence to inform policy decisions Single case study based on interviews with 72 decision-makers from 50 states	Description of research use by phase of policy development, types of research/other information used and how research was used	Backward tracing research use assessment where policy processes were the unit of analysis Compared use of global and local knowledge
*P: Hutchinson et al. 2011* ^[60]^ *(Malawi, Uganda, Zambia)*				
Understand research use in policy decisions	Development of National treatment guidelines for HIV positive TB patients	Overseas Development Institute RAPID Framework (analysis of process; context; evidence and links) Policy case studies from 3 countries Data sources: interviews with policy stakeholders; document analysis	Description of key research and policy events Explanation of the uptake and use of research based on context, evidence and links	Backward tracing research use assessment where policy processes were the unit of analysis Used an across country comparison to examine how context influences policy development
*Q: Lavis et al. 2003* ^*[74]*^ *(Canada)*				
Understand research use in policy processes	Development of health service policies in 2 Canadian provinces	No framework used 8 policy case studies (stratified sampling) Data sources: policy-maker interviews; document analysis; survey of research unit-directors (identify local research that was available)	Number of policies in which citable research/other information was used, stage of policy development it was used and examples of how it was used Ways in which policy-makers accessed research	Backward tracing research use assessment where policy processes were the unit of analysis Stratified policy selection by policy type and location
ASSESSMENTS USING ELEMENTS OF FORWARD AND BACKWARD TRACING APPROACHES				
*R: Bunn et al. 2011* ^[11]^ *(United Kingdom)*				
Understand research impact	Nurse home visiting research conducted in the UK UK policy documents relevant to home visiting	Adapted Research Impact Framework (policy impact only) Data sources: content analysis of policy documents; citation analysis of key pieces of research; interviews with prominent researchers about the impacts of United Kingdom home visiting research	Publications cited in policy documents and type of research cited Described examples of policy impact by levels of policy-making, type of policy and nature of policy impact	Backwards and forwards tracing elements Analysis of policy documents compared to information from researchers and citation analysis of research outputs Described by authors as a research impact assessment
*S: Morton 2015* ^*[88]*^ *(United Kingdom)*				
Understand research impact	Research project conducted by the Centre for Research on Families and Relationships and a voluntary organisation (ChildLine Scotland) Development of an alcohol policy at the Scottish Government level	Research Contribution Framework (steps/process of research impact on policy) Single case study Data sources: policy document analysis and policy-maker interviews (policy analysis); interviews/surveys with research partners, end-users and dissemination activity participants/target audience (trace researcher activities and impacts)	Description of the activities and events that led to research impact Description of impacts Effect of context on research impact	Includes backwards and forwards tracing elements Research project is the primary unit of analysis Data from policy analysis triangulated with forward tracing elements of the study Described by author as a research impact assessment
*T: De Goede et al. 2012* ^[19]^ *(Netherlands)*				
Understand research use in policy processes	Local epidemiological research reports published as Local Health Messages Development of local health memoranda	Framework consisting of the research and local health policy context and networks, types of research utilisation, explanations of research use Case studies of the development of Local Health Memoranda in 3 municipalities Data sources: interviews with researchers and key policy actors; survey of other actors; policy document analysis; meeting observation Prospective data collection	Describe process of producing local health messages (research) and local health memorandum (policy) Describe influence of policy-makers beliefs/characteristics on research use Describe the interface between local epidemiologists and local policy actors to explain research use	Includes backwards and forwards tracing elements Focus on interface between development of a specific research output and a related policy

The studies originated from 44 different countries. Three quarters (76%; *n* = 109) were from high-income countries and predominantly the United Kingdom (*n* = 38), the United States of America (*n* = 16), Australia (*n* = 15), and Canada (n = 10). In middle- to low-income countries, a greater number of research use studies than of research impact studies were completed (*n* = 22 vs. *n* = 7, respectively). Most studies (81%; *n* = 116) were published in the last decade (2006–2016). A wide variety of research types and policy decisions were studied, as summarised in Boxes 2 and 3.

### Core elements of the descriptive framework

#### Key drivers and reasons for assessment

The two streams of research were driven by different factors and conducted for different but related reasons. Research impact assessments were primarily driven by pressures to demonstrate that spending money on research is an appropriate use of scarce resources, while research use assessments were primarily driven by a desire to understand and improve the use of research in policy decisions so that health outcomes could be improved. Research impact assessments were most commonly conducted to demonstrate the value of research beyond the academic setting, to identify factors associated with research impact and develop impact assessment methods (Table 3; Fig. 1). Research use assessments were most commonly conducted to understand policy processes and the use of research within them (Table 3; Fig. 1). Intersecting studies were influenced by factors consistent with both streams of research.

**Table 3:** Key drivers and reasons for assessments

Research impact assessments	Research use assessments
*Key factor driving assessments:* Pressures to justify research expenditure by demonstrating that research has benefits beyond academia [5]	*Key factor driving assessments:* Desire to improve policy outcomes through greater use of research in policy decisions (evidence-informed policy movement) [1]
*Reasons for assessment:* *Accountability*:Quantify or describe the nature of research impact usually to demonstrate a return on investment for research funders or programmes of research [17, 21] *Advocacy:* Demonstrate that research investments are worthwhile, current levels of research expenditure are justified or funding for an area of research is warranted or should be increased [1, 17, 21] *Allocate funding:* Decide future research investments based on past research performance [1, 21] *Inform research systems:* Provide information about likelihood of benefit for different types of research or funding strategies to inform future research system organisation [1, 5] *Understand research impact for learning and improvement:* Identify factors associated with impact or lack of impact with a view to understanding how to influence change and develop better ways of delivering research impact [21, 42] *Develop assessment methods:* Test methods of assessment with the goal of developing systems for assessing the benefits from research ^[14, 78]^	*Reasons for assessment:* *Understand research use:* describe nature and type of research use to determine how research is used to inform policy ^[20]^ *Benchmark or audit research use:* Examine the extent of research use in policy-making or by policy agencies: for comparative purposes ^[22, 23]^; to advocate for greater use of research in decision-making ^[40, 68, 143]^; or to evaluate whether policy agencies had achieved their own goals in terms of developing evidence-informed policy ^[21, 33, 126]^ *Understand policy processes:* Understand the many factors, including research, that are considered during policy-making with a view to understanding the role research plays in policy processes ^[129]^ *Understand research translation:* Examine the research policy interface and identify factors associated with research use or lack of use in policy decisions with a view to identify strategies to increase research use ^[13, 19, 38, 139]^

#### Direction of assessment

As depicted in Fig. 1, research impact assessments most commonly used forward tracing approaches (*n* = 61, Table 1; Examples A, F, Table 2), while research use assessments most commonly used backward tracing approaches (*n* = 63; Examples I-Q Table 2). However, there were several groups of studies that deviated from this pattern. Firstly, a few research impact assessments used a backwards tracing approach (*n* = 7; Table 1). For example, starting with a group of related policy documents, tracing backwards from these to identify the use of specific research outputs as an indication of research impact ^[26, 64, 110]^, or tracing the origins of research (country of origin, research funder, type of research) cited in clinical guidelines to identify research that had been impactful ^[41, 70, 76, 77]^ (Example H, Table 2). These backward tracing studies included a systematic analysis of a group of policy documents from a given policy area, rather than examining single policy documents to corroborate claimed impacts, as was common for forward tracing research impact assessments.

Secondly, there were a few studies where the reasons for assessment were more consistent with the research use group, but the study used a forward tracing approach. These studies traced forward from specific research outputs but assessed whether and how these had been used by a specific policy community who had commissioned or were mandated to consider the research under study (*n* = 4, Table 1; Example G Table 2).^[20, 22, 23, 30]^ Individual research user and agency characteristics associated with research use were assessed, as well as the characteristics associated with the research itself. Only policy-makers were interviewed or surveyed, which was unusual for forward tracing assessments, and some assessments involved an element of evaluation or audit of the policy-makers’ responsibilities to consider evidence.

Finally, there was a group of studies sitting within the intersection between the two streams of research that utilised a combination of forward and backward tracing approaches (*n* = 9; Table 1). In some cases, the study authors were explicit about their intentions to utilise both forward and backward tracing approaches to assessment, aiming to triangulate data from both approaches to produce an overall picture of research impact. For example, tracing forward from a programme of research to identify impacts, as well as analysing a group of policy documents to identify how the programme of research had influenced policy ^[11]^, or tracing forward from the activities of researchers to identify impacts as well as analysing a policy process linked to the research ^[88]^ (Examples R, S, Table 2). These studies drew mainly on elements consistent with the research impact literature. Other intersecting studies were more difficult to classify as they focussed on the interface between a specific research output and a specific policy outcome, examining both the research production and dissemination process as well as the policy decision-making process (Example T, Table 2) ^[19, 31, 63, 127]^.

#### Unit of analysis

The unit of analysis for studies starting with research ranged from discrete research projects with a defined start, end-point and limited set of findings, to increasingly larger programmes of work, representing multiple research studies linked through the researcher, research topic area or research funder. Thus, we classified studies (Fig. 1; Table 4) in terms of whether the unit of analysis was a research project (Examples A, B, Table 2), programme of research (Example D, Table 2), research centre (Example E, Table 2), or portfolio of research (Example F, Table 2). Research projects were the most common unit of analysis (*n* = 52; Table 1).

The unit of analysis for assessments starting with a policy outcome included (Fig. 1; Table 4) a group of policy documents or process for developing a specific document/s (Examples H–J, Table 2), decision-making committees where the committee itself and the decisions made over a period of time were under study (Examples K–L, Table 2), and policy processes where a series of events and debate over time, culminating in a decision to implement or reject a course of policy action, was studied (Examples M–Q, Table 2). Policy processes were the most common unit of analysis (*n* = 49; Table 1).

**Table 4:** Units of analyses for included studies

Assessments starting with research	Assessments starting with a policy outcome
*Research project*: Research conducted by a single research team. Has a defined start and end-point and limited set of research findings [17]. Basic unit of funding for most research funding schemes. Data is collated on a per project basis. Impacts from multiple projects may be summed to provide an overall estimate of impact for a portfolio of research *Programme of research:* Multiple research projects linked by research topic area (different researchers/research teams) or through the lead investigator or research team conducting the research (common topic area and research team). Impacts reported in relation to the entire programme rather than in relation to individual research projects or outputs *Research centre:* Research conducted by a group of researchers linked through common administrative processes and related research activity. Includes analysis of the broader long-term contribution of the centre as a whole, usually combined with analysis of individual research projects or programmes of research conducted by the centre ^[51, 115]^ *Research portfolio:* Research funded under a single funding stream where the assessment includes an analysis of overall awareness and use amongst end-users of any research from the portfolio as a whole (as opposed to awareness and use of specific research outputs). Can be combined with analysis of funded research projects or programmes of research ^[78, 99]^	*Policy documents:* Content of policy documents or process for developing a specific policy document/s examined. Data is collated on a per document basis. Includes an analysis of all policy documents or a sample of policy documents within a policy area as opposed to examining a single policy document to corroborate a claimed impact. Policy documents represent the end-point (outputs) of policy deliberations or the policy position at a given point in time. Focus is on discrete policy outputs rather than the extended policy process they may be part of ^[74]^ *Committee/expert groups:* Process of decision-making of a specific committee or expert group, over a period of time, is analysed ^[24, 137]^. The committee itself is the unit of analysis rather than the policy documents produced by the committee, although these may be analysed as part of the assessment *Policy process:* Analysis of a series of events/decisions, made over a period of time culminating in a policy outcome [62]. May examine the overall policy process or divide the analysis according to stages in the policy process (e.g. pre-policy/awareness raising; policy formulation/development; post-policy enactment/implementation) ^[74]^

Several studies compared the impacts of different types of research grants (e.g. project, fellowship, research centre grants) and thus included more than one unit of analysis ^[14, 55, 141]^. The same was true for studies adopting both forward and backwards tracing approaches, where the starting point for assessment was both a specific research project or programme and a specific policy outcome or process ^[11, 19, 31, 63, 88, 127]^.

#### Theories and conceptual models underpinning assessments

It was common for studies in our sample to draw on existing models and theories of research use and policy-making [1, 11, 12]. These were used to form conceptual frameworks and organise assessments and discussions around the nature of research use or impacts identified in assessments. As well as drawing on this broad base of literature, the studies often utilised a specific framework to structure data collection, analysis and to facilitate comparisons between cases (Fig. 1). Specific frameworks were more often utilised in research impact assessments than research use assessments (*n* = 46 vs. *n* = 23, respectively; Table 1).

The frameworks in the set of research impact assessments most commonly provided a structure for examining multiple categories or types of impact, and sometimes included more detailed case studies of how and why impacts occurred. The Payback Framework [30] was the most commonly used framework of this nature (*n* = 23). The elements and categories of the Payback Framework seek to capture the diverse ways in which impacts arise, including the interactions between researchers and end-users across different stages of the research process and feedback loops connecting stages [6, 20]. Other similar frameworks included the Research Impact Framework [31], the Canadian Academy of Health Sciences impact framework [32] or frameworks that combined these existing approaches ^[11, 16, 85]^. In addition, some studies used frameworks based on a logic model approach to describe the intended outputs, outcomes and impacts of a specific portfolio of research, sometimes including multiple categories of impact ^[44, 78, 98, 114]^ or focussing on policy impacts alone ^[99, 100]^. Finally, there were several examples of studies utilising frameworks based on contribution analysis, an approach to exploring cause and effect by assessing the contribution a programme is making to observed results [33]. Such frameworks emphasise the networks and relationships associated with research production and focus on the processes and pathways that lead to impact rather than outcomes [27, 33]. Examples included frameworks that prompted the evaluation of research dissemination activities to measure changes in awareness, knowledge, attitudes and behaviours of target audiences as precursors to impact ^[88]^; models that focussed on actor scenarios or productive interactions prompting the examination of the pathways through which actors linked to research, and distal to it, took up the research findings to describe impact (Contribution Mapping [34]) ^[57, 69, 87]^; and frameworks that prompted an analysis of network interactions and flows of knowledge between knowledge producers and users ^[84]^. Most frameworks utilised in research impact assessments depicted a linear relationship between research outputs and impacts (that is, simple or direct links from research to policy), albeit with feedback loops between policy change and knowledge production included. Research impact studies rarely utilised frameworks depicting the relationship between contextual factors and research use ^[84, 133]^.

By contrast, contextual factors featured strongly in the models and frameworks utilised in the research use assessments examined. Research use frameworks most commonly provided a mechanism for understanding how issues entered the policy agenda or how policy decisions were made. Dynamic and multidirectional interactions occurring between the policy context, actors and the evidence were emphasised, thus providing a structure for examining the factors that were influential in the policy process. Many examples were utilised, including Kingdon’s Multiple Streams Theory [35], Walt and Glison’s Health Policy Analysis Triangle [36], Dobrow’s framework for context-based evidence-based decision-making [37], Lomas’s framework for contextual influences on the decision-making process [26], and the Overseas Development Institutes Research and Policy in Development (RAPID) Framework [38]. In addition, models provided a structure for analysis of different stages of the policy process ^[2, 61, 122]^ or according to different types of research use (e.g. conceptual, symbolic, instrumental research use ^[19]^, research use continuum [11]) ^[4, 61]^. Finally, evidence typologies were sometimes used to structure assessments, so the use of research evidence could be compared to the use of information from other sources ^[9, 143]^.

Intersecting studies utilised frameworks that focussed on the research–policy interface depicting the links or interactions occurring between researchers and policy-makers during research and policy development ^[19, 124, 129]^. There were also examples of models depicting channels for knowledge diffusion ^[63, 127]^.

#### Methods of assessment

##### Data sources

We found that similar data sources were used in both research impact and research use assessments (Fig. 1), including interviews, surveys, policy documents, focus groups/discussion groups, expert panels, literature reviews, media sources, direct observation and bibliometric data. Research impact assessments also utilised healthcare administrative data ^[64, 78, 119]^ and routinely collected research impact data (e.g. ResearchFish [39] ^[43]^, Altmetrics [40] ^[12]^, UK Research Excellence Framework case studies [41] ^[42]^).

##### Triangulation of data and case studies

Most studies triangulated data from multiple sources, often in the form of case studies (Table 1). Research use assessments were more likely to describe single case studies than research impact assessments, where multiple case study designs were more common (Table 1). Data was most commonly sourced from a combination of interviews, surveys and document analysis, for research impact assessments, and interviews and document analysis for research use assessments. Research impact assessments often combined a larger survey with a smaller number of case studies, to obtain breadth as well as depth of information. Surveys were rarely used in research use assessments ^[22, 23, 33, 74, 135]^.

Cases for both research impact and research use studies were most often purposely selected, based on the likelihood of having impacts for research impact assessments and known research use or to illustrate a point (e.g. delay in research uptake, influence of various actors) for research use assessments. Exceptions included assessments where a whole of sample ^[16, 85, 95, 115]^ or stratified sampling approach ^[37, 54, 66, 74, 107, 140]^ was adopted.

##### Scoring of impacts and research use

In some research impact and research use assessments, a process for scoring impacts or research use was utilised, usually to compare cases ^[2, 7, 10, 14, 16, 17, 37, 42, 50, 54, 62, 64, 79, 90, 91, 97, 107, 113, 115, 117, 140, 141]^. Examples of the scoring criteria used for each group are provided in Table 5.

**Table 5:** Scoring criteria utilised in research impact and research use assessments

Research impact assessments	Research use assessments
*Reach* of impact amongst end-users (e.g. local uptake vs. international/multi-country impact) ^[16, 42, 54]^	*Degree of influence* of research on the policy decision in relation to other factors and/or other sources of information ^[37, 74]^ [1]
*Importance/significance* of the policy change (e.g. the degree to which the policy change is likely to lead to improved health outcomes) ^[16, 42, 54]^	*Consistency* between the available evidence and the policy decision (i.e. the degree to which the policy decision reflects the available evidence) ^[90, 91, 113]^
*Degree of the influence/attribution* of the research on the policy outcome in relation to other factors and sources of information ^[16, 42, 54]^	*Quality* of research used to inform the decision (e.g. critical appraisal of the quality and type of research used) ^[62]^
*Corroboration* of the claimed impact (e.g. degree to which the claim is supported by evidence from policy documents or decision-makers) ^[16]^	*Extent of research use* compared to what was available for use ^[74, 131]^
	*Requirements for use* compared to actual use ^[117, 130]^

##### Study participants

Where respondents were surveyed or interviewed, research impact assessments tended to focus on the perspectives of researchers (Table 1), most commonly questioning researchers about the impacts of their own research and end-users directly linked to the research or researchers under study. Research use assessments tended to draw on the views of a wider range of stakeholders (Table 1), and where researchers were interviewed, they were often interviewed as experts/advisors rather than about the role played by their own research.

##### Data analysis methods used

As most of the data collected in both research impact and research use studies was qualitative in nature, qualitative methods of analysis or basic descriptive statistics were most commonly used. However, there were studies in which more complex statistical analyses of quantitative data were employed. For example, logistic or linear regression analyses to determine which variables were associated with research impact ^[73, 140]^, research use by policy-makers ^[20, 22, 23]^ or policy decision-making ^[17, 79]^. In addition, one study used network analysis to explore the nature and structure of interactions and relationships between the actors involved in policy networks ^[118]^

##### Retrospective versus prospective data collection

Most assessments collected data retrospectively (i.e. sometime after the research findings were available (from 2 to 20 years) for forward tracing assessments, or after a policy outcome had occurred for backwards tracing assessments). Prospective data collection was rare (i.e. data collected during and immediately after research completion for forward tracing assessments, or during policy development for backwards tracing assessments) ^[19, 49, 103, 137]^.

#### End-point for assessment

Depending on the starting point for assessment, the end-point of assessment was either to describe policy impact or research use (Fig. 1). Intersecting studies reported how specific research was used in relation to a specific policy outcome. Definitions for what constituted a ‘policy impact’ or ‘research’ in the assessment differed between studies.

##### Definitions of policy impact

For studies commencing with research, not all studies explicitly defined what was considered a policy impact, rather describing any changes attributable to the research. Where definitions were provided, some definitions required evidence of the explicit application of research in policy decisions; that is, the research directly influenced the policy outcome in some way ^[16]^. There were also examples where incremental steps on the pathway to policy impact, such as changes in policy-makers’ awareness and knowledge or process measures (e.g. interaction (participation on an advisory committee), dissemination (presentation of research findings to policy-makers)) ^[88]^, were counted as impacts. Here, such process measures were seen as “*a more practical way of looking at how research interacts with other drivers to create change*” rather than looking “*for examples of types of outputs or benefits to specific sectors*” ([42] p.12). In addition, a shift in language and focus from ‘attribution’ to ‘contribution’ was promoted by some authors to suggest that research was only one factor amongst many influencing outcomes ^[57,69,87,88]^. Some studies reported policy impacts alone, while others reported multiple categories of impact. Where multiple categories of impact were reported, impacts were not always categorised in the same way so that what was considered a policy impact in one study would have fallen under a different category of impact in another (e.g. policy impact vs. health services impact) ^[16, 114, 140]^.

##### Definitions of research

Conversely, for studies commencing with a policy outcome, not all studies provided a definition for what constituted ‘research’ in the assessment, rather summarising the relevant scientific literature to provide an overview of the research available to policy-makers.^[8, 13, 18]^ Where definitions were provided, some studies used narrower definitions of research, such as ‘citable’ academic research only ^[74]^, as opposed to broader definitions where ‘any data’ that played a role in shaping/driving policy change was included in the definition of research.^[4]^ Other authors defined a specific type of research to be identified in the assessment (e.g. economic analyses ^[49, 103, 137]^, research on patient preferences ^[130]^, evidence of effect and efficiency ^[37, 101]^). Most authors of research use studies explicitly recognised that research was only one source of information considered by policy-makers. Some studies explored the use of various types of information (e.g. contextual socio-political, expert knowledge and opinion, policy audit, synthesis, reviews, economic analyses ^[9]^), as well as research (e.g. scientific literature ^[9]^). In addition, some studies included research in a broader definition of ‘evidence’ alongside other information sources (e.g. including research study results, findings on monitoring and evaluation studies and population-based surveys, Ministry of Health reports, community complaints and clinical observations as ‘evidence’ used during policy-making ^[90]^). Finally, there were examples of research being distinguished in terms of local and international research sources ^[8, 61]^.

#### Common outcomes reported

Despite differing trajectories of assessments, research impact and research use assessments reported similar types of outcomes (Fig. 1), although the discussion was framed in different ways. For example, qualitative methods were utilised in both research impact and research use assessments to describe the impacts that occurred or how research had been used. Authors from both groups described outcomes in terms of conceptual, symbolic or instrumental uses of research ^[4, 19, 20, 44, 61, 72, 74, 129, 133, 143]^, direct/explicit and indirect impacts/uses of research ^[42, 74, 87]^, or research use according to the stage of the policy process at which the use occurred ^[61, 75, 85, 100, 122]^ (Box 4). Other assessments adopted a quantitative approach, to sum impacts or research use across units of analysis, resulting in an overall measure of impact for a research portfolio or area of research ^[50, 55, 73, 141]^, or in policy domains as a benchmark of research use for that policy area ^[40, 68, 143]^.

In tackling the question about what is needed to facilitate research utilisation and research impact, both research impact and research use studies reported on the processes and pathways through which research was utilised and the factors associated with research use. Studies from both groups also focussed on the role played by various actors in the policy process. Research impact assessments tended to focus on research and researchers as facilitators of impact, commonly examining the dissemination, engagement activities, networks and other characteristics of specific researchers in considering impact pathways and factors associated with impact. Study participants were usually linked to the research or researchers under study in some way and commonly provided a perspective about the context surrounding the uptake of the research under study, rather than being asked about the policy context more broadly (e.g. sociocultural, political, economic factors and other information sources influencing the policy process).

In contrast, research use assessments generally examined the role played by a wide range of actors in the policy process (e.g. politicians, policy-makers, service providers, donors, interest groups, communities, researchers) and links between the research and policy interface (e.g. networks, information exchange activities, capacity-building activities, research dissemination activities, partnerships). Variables associated with policy-makers and policy organisations (e.g. culture, ideologies, interests, beliefs, experience), as well as the research and researchers, were examined. In addition, assessments tended to adopt a broader approach when examining the policy context, considering research along-side a range of other influencing factors.

## Discussion

In this paper, we provide a framework for categorising the key elements of two parallel and sometimes intersecting streams of research – studies assessing the policy impacts of research and studies assessing research use in policy processes. Within the studies examined, research impact assessments were primarily conducted to demonstrate the value of research in terms of producing impacts beyond the academic setting. This information was important for grant funding bodies seeking to account for research expenditure. As such, research impact assessments focussed on research, identifying impacts that could be attributed to specific research projects or programmes and the mechanisms or factors associated with achieving these impacts. Such studies predominantly used forward tracing approaches, where research projects (the most common unit of grant funding) were the unit of analysis. Research use assessments, on the other hand, were conducted with a view to improving policy outcomes by identifying ways in which research use could be enhanced. Here, the assessments most commonly focussed on understanding policy processes, whether and how research was used and the mechanisms and factors that facilitated research use. Thus, backward tracing approaches predominated; starting with a specific policy outcome and utilising a policy analysis frame to consider the influence of research alongside other factors. The approaches to assessment influenced the nature of the findings, so their respective strengths and limitations should be considered.

### Strengths and limitations of approaches

The main difference between the research impact and research use studies we considered was the relative focus on the influence of ‘specific research’ in relation to a policy outcome. Research impact assessments focused on specific pieces or bodies of research so that observed effects could be linked to grant funding, researchers or research groups [17]. While research projects were most commonly assessed, we encountered examples where the unit of analysis was broadened to include larger programmes of research, in some respects to overcome problems attributing impacts to single projects within a researcher’s larger body of work. However, this did not overcome problems separating the influence of this research from that conducted by others in the same field [46, 47]. Broadening the unit of analysis also created problems with defining the scope of assessment in terms of where the programme of research started and ended, as research generally builds on earlier research and itself [46, 47]. In addition, the larger the programme of research under study, the more diffuse its impacts became, making them more difficult to identify and attribute to individuals or groups of researchers and certainly funding bodies [48–50].

The research use assessments on the other hand, tended to examine the role played by research in more general terms rather than attempting to determine the contribution made by specific research projects or programmes. Indeed, such assessments often highlighted the relationships between related or conflicting programmes of research, local and international research and other sources of information (e.g. expert opinion, practice-based knowledge). There were also examples of research use assessments that examined the use of ‘evidence’ without separating the influence of research from other information sources (e.g. scientific research, population surveys, administrative data and reports, community complaints, clinical/expert opinion). These differences raise the issue about whether a single research project is a valid unit of analysis [17, 26] and what unit of analysis is the most appropriate. While it might be useful to focus on specific research for research accountability purposes and ease of measurement, the use of information assimilated from multiple sources is consistently reported as closer to the reality of how knowledge enters the policy debate and contributes to policy outcomes [45].

Different approaches to assessment will also give rise to a differential emphasis on the role of research in policy-decisions and the relevance of context [27, 42]. The research impact assessments we examined tended to focus on why impacts occurred (did not occur) and the contextual factors associated with research uptake, rather than adopting a wider frame to examine other factors and information sources that may have been influential. Focusing on research uptake may mean that details of the policy story are missed and the influence of the research overstated [17], whereas research use assessments commonly sought to understand the relationship between the various factors involved in decision-making and the role played by research within this mix. Tracing backwards to examine the policy process in this way is likely to provide a more accurate picture of research influence [11]. However, this finding depended on the unit of policy analysis chosen for assessment. As policy decisions often build on previous policy decisions which in turn may be influenced by research [29], focussing on a narrow aspect of the policy process as a unit of analysis may not capture all of the research considered in reaching an outcome or the full range of factors that may have influenced the policy decision [51]. In particular, policy documents represent the outputs of policy discussions or the policy position at a single point in time, so examining research use at this level may mean that it is missed, or undue emphasis is placed on the influence of cited research [51].

As well as the relative emphasis placed on research, the assessment approach itself may determine the type and nature of impacts or research use identified. For example, it was common for the research impact assessments we examined to seek evidence linking the research in question to the policy outcome (e.g. seeking corroborating testimony from policy-makers or evidence in policy documents). Studies also sometimes sought to quantify the strength of this relationship, or the relative contribution of the research in relation to other factors, by subjectively scoring the extent of research influence on the policy outcome. This focus on measurable links between research and policy that can be proven meant that such assessments were more likely to identify instances where research had been directly applied in policy discussions (instrumental uses) [52]. In addition, the research impact assessments we examined most commonly utilised frameworks suggesting direct and linear links between research and policy (albeit with feedback loops included), and thus potentially overlooking indirect or conceptual uses. Finding evidence for indirect influences, such as changes in awareness and understanding of an issue, may be challenging [27]. To better capture indirect and as well as direct impacts, some authors propose that research impact should be measured in terms of the processes (e.g. interactions, dissemination activities) and stages of research adoption amongst end-users/stakeholders resulting from these processes (e.g. changes in awareness, understanding, attitude/perceptions), rather than focussing on outcome-based modes of impact evaluation [27, 34, 42]. This way of thinking about impact helps to identify changes that occur early in the impact pathway and can establish clear links between the research and the contribution it has made [42], however, this may emphasise ‘potential’ rather than actual impact. It can be argued that actual impact only occurs if a stakeholder uses or applies (e.g. to inform or encourage/discourage change) the research results within a policy debate; that is, if there has been a behavioural change because of the knowledge gained [53].

For research use assessments, the nature of research use reported may vary depending on what type of policy process was considered [29]. The studies we examined that assessed specific and discrete policy decisions, for example, committee decisions focussed on making recommendations for practice, also tended to emphasise instrumental research use, as there was a requirement or mandate for research to be directly applied in the decision-making process, whereas studies considering broader policy processes, where events overtime were examined, had the potential to identify the many ways in which research could be utilised. The conceptual models that were adopted in these assessments provided a mechanism for considering how issues entered the policy agenda or how policy decisions were made without a presumption that research had made a direct contribution to the policy outcome. However, assessments of this nature highlighted the difficulties of determining the influence of research on tacit knowledge, where research use lies within other types of information (e.g. expert knowledge) and stakeholder positions [29]. For example, the research use assessments we examined commonly investigated the influence of other information sources and stakeholder’s positions on policy decisions, but stopped short of investigating whether these sources of influence were themselves informed by research [29]. Identifying hidden or unconscious uses of research will always be challenging for both research use and research impact assessments.

Not only does the overall choice of approach influence the assessment findings, but also specific methodological choices. Some methodological issues were common to both research impact and research use assessments. For example, issues to do with the timing of assessment to best capture research impacts or use. In addition, purposeful sampling and the number of case studies conducted influenced how predictive or transferrable the assessment findings were [17, 24, 54]. There were also tensions within both streams between the value of utilising the most comprehensive and robust methods of assessments possible and the resources required for these methods. Case studies, including interviews with study participants, were considered the gold standard method of assessment, but resource intensive to conduct [55]. Policy case studies were particularly time and resource intensive, requiring careful consideration of historical and contextual influences, hence the predominance of single policy case studies amongst the research use assessments we examined [24]. On the research impact side, methods utilising automated data extraction from policy documents and electronic surveys of researchers have been introduced [6, 56]. Such methods are less resource intensive and offer greater potential for implementation on a wide scale, but there is still limited information available about their validity and reliability [5, 6, 57, 58]. There were also instances where methodological choices differed between the two streams of research, influencing outcomes of assessments from each group. For example, researchers or end-users directly associated with the research project or programme under study were most commonly interviewed or surveyed in the research impact assessments, whereas the research use assessments we examined often involved a broader cross section of policy actors and researchers as study participants. These differences provide different perspectives about the role played by research, and thus the method influences the findings.

In essence, the differences between forward and backward tracing assessments highlighted above illustrate how the choices made in assessments alter the phenomenon they aim to examine. In fact, this is similar to other types of evaluation; the assessment process illuminates a particular pathway, perspective or outcome, but another assessment process would see it differently.

### Possibilities for further research

It is likely that the pathways to impact and the degree to which research will be utilised will differ for different types of research and policy areas [1, 29]. Understanding these differences may help researchers and policy-makers to set appropriate goals in terms of research impact and use, as well as to identify the most appropriate pathways through which translation could be achieved. However, we identified only a small number of studies comparing the impacts of different types of research (and only biomedical compared to clinical research) or differences in research use according to policy area. Further studies adopting across-case comparison approaches to investigate these issues would be useful.

In this review, we encountered a lack of consistency in the definitions and terminology applied across the included studies. This was the case for describing the type of research being assessed and what constituted policy impacts in research impact assessments, as well as in defining and categorising forms of evidence and types of policies in research use assessments. Different conclusions about the extent to which policy-making is informed by research may arise from different views about what constitutes research in research use assessments or, conversely, policy impact in research impact assessments [29]. Moving towards the application of consistent definitions across this area of study would also be beneficial [14]. It is also important that authors in future studies are clear about the definitions and ways of thinking about research impact/use applied in assessments, so that comparisons between study findings can be made and limitations made explicit [14].

The two streams of research discussed in this review have developed separately over a similar timeframe. More recently, studies have drawn on elements from both streams of research. Some of these studies are exemplary in many ways, tracing forward from research and backwards from policy to produce case studies which address common limitations in novel and rigorous ways. There is scope for more research impact assessments to borrow from backwards tracing approaches in this way. In addition, very few studies utilising network analyses and applied systems-based theories, were identified in this review. Such approaches may also provide a means of exploring these issues [52].

Most of the studies included in this review appeared to be initiated by researchers for researchers or by research funding bodies. Researchers are now being asked to routinely track the impacts of their own research [6]. This focus on research and researchers places a one-sided emphasis on the role of researchers in getting research into policy. Reducing the waste from research also requires action from policy-makers. Yet, very few studies investigated to what degree the decision-making environment supported research use. To address this imbalance, there is scope for policy agencies to develop mechanisms to assess their own requirements and practices for considering research during policy deliberations, as well as investigating ways to routinely monitor research use.

Finally, there were very few examples of prospective approaches being utilised in either stream of research examined in this review. These approaches have disadvantages, for example, they may not be practical in terms the resources required to trace research or policy processes for extended periods, or it can be difficult to obtain permission to directly observe policy processes or respondents may not be as forthcoming about factors of influence at the time they are occurring (e.g. political debates) [15]. However, prospective approaches to assessment may prompt researchers and end-users to think about research translation from the outset of a research project or policy process, and provide opportunities for appropriate and tailored translational interventions to be embedded into work processes [59]. Routine data collection and, in particular, process metrics related to research translation activities could be used to provide feedback about areas requiring attention in order to improve research uptake [59]. With the advent of routine data collection systems, the potential advantages of this approach could be explored in future studies.

#### Limitations of this review

This review only included English language publications and therefore studies from non-English speaking countries will be under-represented. This may in part explain our findings around the high proportion of studies conducted in high-income countries. The studies included in this review are likely to be broadly representative of the type of studies conducted to date. However, due to our exclusion criteria, we may have missed examples of studies published only in the grey literature or methodological approaches that have not been empirically tested. For example, we identified only a small number of peer-reviewed publications where a programme of research was the unit of analysis. The preparation of case studies based on a researcher’s programme of research was adopted in both the Australian Research Quality Framework [60] and more recently the UK Research Excellence framework [41]. Reports describing the application and findings of this approach are available in the grey literature [41, 61]. Finally, author one managed the literature search and inclusion process, as well as extracting primary data from the included articles. This may have introduced some bias, although the other authors of this review were consulted and came to agreement on ambiguous cases. Study authors did not always explicitly describe their studies in terms of the characteristics we have included in our descriptive framework and some studies required judgements to be made regarding classification. Our findings in terms of the number of studies within each category should therefore be considered indicative. However, this issue highlights the need for a framework, such as the one we propose, to facilitate clearer communication about what, in fact, studies were seeking to achieve and how they did it.

## Conclusions

Herein, we have defined the key characteristics of two research streams with the aim of facilitating structured comparisons between studies. In many ways, the separate and distinct development of these two research streams, and their different approach to examining the issues, reflect the much-discussed separation of the two domains of research and policy. The descriptive framework introduced and discussed in this paper provides a ‘missing link’, showing how these two streams intersect, compare and differ. Our framework offers an integrated perspective and analysis, and can be used by researchers to identify where their own research fits within this field of study and to more clearly communicate what is being assessed, how this is done and the limitations of these choices.

We have shown that the approach to assessment can determine the perceived influence of research on policy, the nature of this influence and our understanding of the relationship between research and policy. As such, the two approaches, forward and backward tracing, essentially tell a different story about how (if at all) research-based policy change happens. In some ways, the assessments construct the phenomenon they aim to measure. For example, forward tracing research impact assessments, with their focus on specific research and the activities of researchers, may emphasise direct influences of research on policy and overstate the influence of research in policy processes. Conversely, research use assessments utilising a backwards tracing analysis tend to paint a more complex picture of assimilated knowledge contributing to policy outcomes alongside other influential factors. Combining aspects of the two approaches may provide the best way forward in terms of linking outcomes to specific research, as well as providing a realistic picture of research influence.

## Box 1 Key elements differentiating research impact assessments

• Purpose of assessment [1, 17]

• Type of research or policy assessed [1, 23]

• Direction of analysis (e.g. tracing forwards from research or tracing backwards from a policy outcome) [1, 11, 16, 17]

• Unit of analysis (e.g. whether the analysis starts with a single research project or a broader programme of work) [1, 17]

• Conceptual framework used to organise assessment [1, 6, 11, 16–20, 23, 24]

• Types of outcomes measured (e.g. type/categories of impact and levels of utilisation) [1, 16, 17]

• Methods used to measure outcomes of interest (e.g. data sources, retrospective or prospective data collection; case studies or other methods; scoring impacts) [1, 16, 18, 20, 24]

• Strategies to address attribution of impacts to the research in question [16]

## Box 2 Summary of the types of research under study where research was the starting point for assessment

There was a high degree of variability in the type of research under study, between studies and in some cases within individual studies. The types of research under study differed in terms of topic area (e.g. arthritis research, obesity research, asthma research), discipline (e.g. clinical, public health, health services research), where the research lay along the research continuum (e.g. basic to applied research), and whether the research was primary research or a synthesis of research (e.g. health technology assessments and systematic reviews). It was rare for studies to compare impacts between types of research and only the impacts of biomedical and clinical research were compared in this way ^[25, 140]^. The research under study within individual assessments was most commonly drawn from a single research funder or portfolio of research. Assessments were often commissioned by the government agency ^[1, 14, 34, 39, 43, 54, 67, 69, 73, 78, 82, 84, 87, 93, 98, 107, 114, 115, 121, 123, 132, 144]^, charitable group ^[25, 44, 100, 141]^ or professional body ^[112]^ responsible for funding the research under study.

## Box 3 Summary of the types of policies under study where a policy outcome was the starting point for assessment

Assessments starting with a policy outcome examined a wide range of policies. Policies differed in terms of the policy type (e.g. clinical- and practice-based policies, public health policies, financial and structural policies), topic area (e.g. legislative bills relevant to active living, home nurse visiting, immunisation, malaria prevention, health insurance, drug reimbursement decisions), who was responsible for the final policy decision (e.g. parliament/legislative process, committee or expert group, government department or agency, or local health services), the geographical reach of the policy (e.g. international, national, regional/provincial, or local health policy), the stage or stages of the policy process considered in the assessment (e.g. agenda setting, policy formulation, policy implementation), and whether the decision was to proceed or not with the course of policy action (e.g. ‘go’ or ‘no go’ decisions ^[74]^). There were examples of studies comparing research use for different policy types ^[37, 74, 96, 111, 129, 139]^, at different levels of policy-making ^[13, 102, 111, 137]^, and between different countries ^[45, 48, 83]^. The authors of studies rarely stated if the assessment had been commissioned by the agency responsible for the policy under study ^[21, 33, 74, 126, 135]^.

## Box 4 Common ways of describing use/use

*Conceptual:* Refers to a more general or indirect form of enlightenment where research has an influence on awareness, understanding or attitudes/perceptions amongst policy-makers [29, 43]. Conceptual use of research may influence policy debate (ideas, arguments and criticism), which can then feed forward into policy change [44]. The link between the research and any policy change is indirect but the influence of the research on policy-makers is still tangible and potentially measurable.

*Symbolic:* Where research is used to justify a position or specific action already taken for other reasons or to obtain specific goals based on a predetermined position [29, 44]. This is difficult to measure as policy-makers may not acknowledge or be conscious that they are using research in this way. Therefore, identification of this type of research use may rely on judgement of policy-maker’s intent/motivations for using research.

*Instrumental:* Refers to the explicit application of research to address a policy problem; where research influences issue identification, policy refinement, definition or implementation in a direct and potentially measurable way [29, 44]. That is, policy-makers are aware that they are using research in this way and there may be evidence supporting claimed instances of use.

*Indirect:* Refers to the way in research may enter the policy environment in a diffuse way [44]. Indirect use includes the concept of conceptual use where research results in changes in awareness and knowledge that may subsequently influence policy directions. Here, the change is brought about by research and this is recognised by the research user. Indirect use also includes examples where the influence of research may be unseen and unacknowledged; there is no evidence linking decisions to the findings of research, yet a linkage of some sort seems to have existed [27, 29]. This type of indirect influence of research may be exerted on policy decisions through socially shared ‘tacit knowledge’ (e.g. expert opinion, public perception or practice-based knowledge) or through stakeholder positions [29].

*Direct:* Refers to the explicit or direct application of research to policy. Sometimes used inter-changeably with instrumental use.

*Stages of policy development:* Research use described according to the stages of policy development, which vary between models but commonly include identification, agenda-setting, consideration of potential actions/policy formulation, implementation and evaluation [45]

## Additional file

Additional file 1: List of included empirical studies. (DOCX 40 kb)
