# Supplementary material for: Looking both ways: a review of methods for assessing research impacts on policy and the policy utilisation of research
Source: Health Res Policy Syst. 2018 Jun 25;16:54. doi: 10.1186/s12961-018-0310-4 (PMC6019310; doi:10.1186/s12961-018-0310-4)
Supplement: Supplementary file 1 — List of included empirical studies. (DOCX 40 kb) [file 12961_2018_310_MOESM1_ESM.docx]

**Additional file 1**

**List of included empirical studies**

1. Adam P, Solans-Domènech M, Pons JMV, Aymerich M, Berra S, Guillamon I, Sánchez E, Permanyer-Miralda G: **Assessment of the impact of a clinical and health services research call in Catalonia**. *Research Evaluation* 2012, **21**(4):319-328. <http://dx.doi.org/10.1093/reseval/rvs024>

2. Agyepong IA, Adjei S: **Public social policy development and implementation: a case study of the Ghana National Health Insurance scheme**. *Health Policy Plan* 2008, **23**(2):150-160. <http://dx.doi.org/10.1093/heapol/czn002>

3. Albert MA, Fretheim A, Maiga D: **Factors influencing the utilization of research findings by health policy-makers in a developing country: The selection of Mali's essential medicines**. *Health Research Policy and Systems* 2007, **5** (2). <http://dx.doi.org/10.1186/1478-4505-5-2>

4. Allen ST, Ruiz MS, O'Rourke A: **The evidence does not speak for itself: The role of research evidence in shaping policy change for the implementation of publicly funded syringe exchange programs in three US cities**. *Int J Drug Policy* 2015, **26**(7):688-695. <https://dx.doi.org/10.1016/j.drugpo.2015.04.008>

5. Anderson R: **The policy impact of population health surveys: an illustration of the measurement challenges using the NSW Health Survey**. *Evidence & Policy: A Journal of Research, Debate and Practice* 2006, **2**(2):167-183. <https://dx.doi.org/10.1332/174426406777068975>

6. Apollonio DE, Bero LA: **Evidence and argument in policymaking: development of workplace smoking legislation**. *BMC Public Health* 2009, **9**:189. <https://dx.doi.org/10.1186/1471-2458-9-189>

7. Aymerich M, Carrion C, Gallo P, Garcia M, Lopez-Bermejo A, Quesada M, Ramos R: **Measuring the payback of research activities: a feasible ex-post evaluation methodology in epidemiology and public health**. *Social science & medicine (1982)* 2012, **75**(3):505-510. <https://dx.doi.org/10.1016/j.socscimed.2012.03.044>

8. Blume S, Tump J: **Evidence and policymaking: The introduction of MMR vaccine in the Netherlands**. *Social science & medicine (1982)* 2010, **71**(6-3):1049-1055. <https://dx.doi.org/10.1016/j.socscimed.2010.06.023>

9. Bowen S, Zwi AB, Sainsbury P, Whitehead M: **Killer facts, politics and other influences: what evidence triggered early childhood intervention policies in Australia?** *Evidence & Policy* 2009, **5**. 10.1332/174426409X395394 <https://doi.org/10.1332/174426409X395394>

10. Brambila C, Ottolenghi E, Marin C, Bertrand JT: **Getting results used: evidence from reproductive health programmatic research in Guatemala**. *Health Policy Plan* 2007, **22**(4):234-245. <https://dx.doi.org/10.1093/heapol/czm013>

11. Bunn F, Kendall S: **Does nursing research impact on policy? A case study of health visiting research and UK health policy**. *Journal of Research in Nursing* 2011, **16**(2):169-191. <https://dx.doi.org/10.1177/1744987110392627>

12. Bunn F, Trivedi D, Alderson P, Hamilton L, Martin A, Iliffe S: **The impact of Cochrane Systematic Reviews: A mixed method evaluation of outputs from Cochrane Review Groups supported by the UK National Institute for Health Research**. *Systematic Reviews* 2014, **3**(1). <http://dx.doi.org/10.1186/2046-4053-3-125>

13. Burris H, Parkhurst J, Adu-Sarkodie Y, Mayaud P: **Getting research into policy - Herpes simplex virus type-2 (HSV-2) treatment and HIV infection: international guidelines formulation and the case of Ghana**. *Health Research Policy and Systems* 2011, **9**(1):S5. 10.1186/1478-4505-9-S1-S5 <http://dx.doi.org/10.1186/1478-4505-9-S1-S5>

14. Buxton M, Hanney S, Packwood T, Roberts S, Youll P: **Getting Reearch into Practice: Assessing Benefits from Department of Health and National Health Service Research & Development**. *Public Money and Management* 2000, **20**(4):29-34. <https://dx.doi.org/10.1111/1467-9302.00233>

15. Caddell AJ, Hatchette JE, McGrath PJ: **Examining the impact of health research facilitated by small peer-reviewed research operating grants in a women's and children's health centre**. *BMC research notes* 2010, **3**(1):107. https://dx.doi.org/[10.1186/1756-0500-3-107](https://dx.doi.org/10.1186%2F1756-0500-3-107)

16. Cohen G, Schroeder J, Newson R, King L, Rychetnik L, Milat AJ, Bauman AE, Redman S, Chapman S: **Does health intervention research have real world policy and practice impacts: testing a new impact assessment tool**. *Health Research Policy and Systems* 2015, **13**(1):3. <https://dx.doi.org/10.1186/1478-4505-13-3>

17. Dakin HA, Devlin NJ, Odeyemi IA: **"Yes", "No" or "Yes, but"? Multinomial modelling of NICE decision-making**. *Health Policy* 2006, **77**(3):352-367. <https://dx.doi.org/10.1016/j.healthpol.2005.08.008>

18. Daniels K, Lewin S: **Translating research into maternal health care policy: a qualitative case study of the use of evidence in policies for the treatment of eclampsia and pre-eclampsia in South Africa**. *Health Res Policy Syst* 2008, **6**:12. <https://dx.doi.org/10.1186/1478-4505-6-12>

19. De Goede J, Putters K, van Oers HAM: **Utilization of epidemiological research during the development of local public health policy in the Netherlands: a Case Study Approach**. *Social Science & Medicine* 2012, **74**. <https://doi.org/10.1016/j.socscimed.2011.11.014>

20. de Goede J, van Bon-Martens MJ, Mathijssen JJ, Putters K, van Oers HA: **Looking for interaction: quantitative measurement of research utilization by Dutch local health officials**. *Health Research Policy and Systems* 2012, **10**(1):9. <http://dx.doi.org/10.1186/1478-4505-10-9>

21. Deas L, Mattu L, Gnich W: **Intelligent policy making? Key actors' perspectives on the development and implementation of an early years' initiative in Scotland's public health arena**. *Social Science & Medicine* 2013, **96**:1-8. <https://dx.doi.org/10.1016j.socscimed.2013.07.001>

22. Dobbins M, Cockerill R, Barnsley J, Ciliska D: **Factors of the innovation, organization, environment, and individual that predict the influence five systematic reviews had on public health decisions**. *Int J Technol Assess Health Care* 2001, **17**(4):467-478.

23. Dobbins M, Thomas H, O'Brien MA, Duggan M: **Use of systematic reviews in the development of new provincial public health policies in Ontario**. *Int J Technol Assess Health Care* 2004, **20**(4):399-404.

24. Dobrow MJ, Goel V, Lemieux-Charles L, Black NA: **The impact of context on evidence utilization: A framework for expert groups developing health policy recommendations**. *Social Science and Medicine* 2006, **63**(7):1811-1824. <http://dx.doi.org/10.1016/j.socscimed.2006.04.020>

25. Donovan C, Butler L, Butt AJ, Jones TH, Hanney SR: **Evaluation of the impact of National Breast Cancer Foundation-funded research**. *Medical Journal of Australia* 2014, **200**(4):214-218. <http://dx.doi.org/10.5694/mja13.10798>

26. Drew CH, Pettibone KG, Finch FO, Giles D, Jordan P: **Automated Research Impact Assessment: A New Bibliometrics Approach**. *Scientometrics* 2016, **106**(3):987-1005. <http://dx.doi.org/10.1007/s11192-015-1828-7>

27. Duke K, Thom B: **The role of evidence and the expert in contemporary processes of governance: the case of opioid substitution treatment policy in England**. *Int J Drug Policy* 2014, **25**(5):964-971. <http://dx.doi.org/10.1016j.drugpo.2014.01.015>

28. El-Jardali F, Bou-Karroum L, Ataya N, El-Ghali HA, Hammoud R: **A retrospective health policy analysis of the development and implementation of the voluntary health insurance system in Lebanon: learning from failure**. *Social Science & Medicine* 2014, **123**:45-54. <http://dx.doi.org/10.1016j.socscimed.2014.10.044>

29. El-Jardali F, Hammoud R, Younan L, Nuwayhid HS, Abdallah N, Alameddine M, Bou-Karroum L, Salman L: **The making of nursing practice law in Lebanon: a policy analysis case study**. *Health Res Policy Syst* 2014, **12**:52. <http://dx.doi.org/10.1186/1478-4505-12-52>

30. Elliott H, Popay J: **How are policy makers using evidence? Models of research utilisation and local NHS policy making**. *Journal of Epidemiology and Community Health* 2000, **54**(6):461. <http://jech.bmj.com/content/54/6/461.abstract>

31. Ensor T, Clapham S, Prasai DP: **What drives health policy formulation: Insights from the Nepal maternity incentive scheme?** *Health Policy* 2009, **90**(2):247-253. <http://dx.doi.org/10.1016/j.healthpol.2008.06.009>

32. Etiaba E, Uguru N, Ebenso B, Russo G, Ezumah N, Uzochukwu B, Onwujekwe O: **Development of oral health policy in Nigeria: an analysis of the role of context, actors and policy process**. *BMC oral health* 2015, **15**:56. <http://dx.doi.org/10.1186/s12903-015-0040-8>

33. Evans BA, Snooks H, Howson H, Davies M: **How hard can it be to include research evidence and evaluation in local health policy implementation? Results from a mixed methods study**. *Implementation Science* 2013, **8**:17. <http://dx.doi.org/10.11861748-5908-8-17>

34. Ferguson B, Kelly P, Georgiou A, Barnes G, Sutherland B, Woodbridge B: **Assessing payback from NHS reactive research programmes**. *Journal of management in medicine* 2000, **14**(1):25-36. <http://dx.doi.org/10.1108/02689230010340363>

35. Flitcroft KL, Salkeld GP, Gillespie JA, Trevena LJ, Irwig LM: **Fifteen years of bowel cancer screening policy in Australia: putting evidence into practice?** *Medical Journal of Australia* 2010, **193**(1):37-42. <https://www.mja.com.au/journal/2010/193/1/fifteen-years-bowel-cancer-screening-policy-australia-putting-evidence-practice>

36. Florin D: **Scientific uncertainty and the role of expert advice: the case of health checks for coronary heart disease prevention by general practitioners in the UK**. *Social science & medicine (1982)* 1999, **49**(9):1269-1283. <https://doi.org/10.1016/S0277-9536(99)00165-3>

37. Frey K, Widmer T: **Revising Swiss Policies: The Influence of Efficiency Analyses**. *American Journal of Evaluation* 2011. <https://doi.org/10.1177/1098214011401902>

38. Gilson L, McIntyre D: **The interface between research and policy: Experience from South Africa**. *Social Science & Medicine* 2008, **67**(5):748-759. <http://dx.doi.org/10.1016/j.socscimed.2008.02.005>

39. Gold M, Taylor EF: **Moving research into practice: lessons from the US Agency for Healthcare Research and Quality's IDSRN program**. *Implementation Science* 2007, **2**(1):9. 10.1186/1748-5908-2-9 <http://dx.doi.org/10.1186/1748-5908-2-9>

40. Gollust SE, Kite HA, Benning SJ, Callanan RA, Weisman SR, Nanney MS: **Use of research evidence in state policymaking for childhood obesity prevention in Minnesota**. *American Journal of Public Health* 2014, **104**(10):1894-1900.<https://doi.org/10.2105AJPH.2014.302137>

41. Grant J, Cottrell R, Cluzeau F, Fawcett G: **Evaluating “payback” on biomedical research from papers cited in clinical guidelines: applied bibliometric study**. *Bmj* 2000, **320**(7242):1107-1111. <https://doi.org/10.1136/bmj.320.7242.1107>

42. Greenhalgh T, Fahy N: **Research impact in the community-based health sciences: An analysis of 162 case studies from the 2014 UK Research Excellence Framework**. *BMC Medicine* 2015, **13 (1) (no pagination)**(232). <https://doi.org/10.1186/s12916-015-0467-4>

43. Guthrie S, Bienkowska-Gibbs T, Manville C, Pollitt A, Kirtley A, Wooding S: **The impact of the National Institute for Health Research Health Technology Assessment programme, 2003-13: a multimethod evaluation**. *Health Technol Assess* 2015, **19**(67):1-291. <https://doi.org/10.3310/hta19670>

44. Gutman MA, Barker DC, Samples-Smart F, Morley C: **Evaluation of Active Living Research progress and lessons in building a new field**. *American journal of preventive medicine* 2009, **36**(2 Suppl):S22-33. <https://doi.org/10.1016/j.amepre.2008.10.009>

45. Haas M, Ashton T, Blum K, Christiansen T, Conis E, Crivelli L, Lim MK, Lisac M, Macadam M, Schlette S: **Drugs, sex, money and power: an HPV vaccine case study**. *Health Policy* 2009, **92**(2-3):288-295. <https://doi.org/10.1016/j.healthpol.2009.05.002>

46. Hailey D: **A preliminary survey on the influence of rapid health technology assessments**. *International Journal of Technology Assessment in Health Care* 2009, **25**(3):415-418. <https://doi.org/10.1017/s0266462309990067>

47. Hailey D, Corabian P, Harstall C, Schneider W: **The use and impact of rapid health technology assessments**. *Int J Technol Assess Health Care* 2000, **16**(2):651-656.

48. Hamalainen RM, Aro AR, van de Goor I, Lau CJ, Jakobsen MW, Chereches RM, Syed AM, Consortium R: **Exploring the use of research evidence in health-enhancing physical activity policies**. *Health Research Policy & Systems* 2015, **13**:43. <https://dx.doi.org/10.1186/s12961-015-0047-2>

49. Hammad EA: **The Use of Economic Evidence to Inform Drug Pricing Decisions in Jordan**. *Value in Health*, **19**(2):233-238. <https://dx.doi.org/10.1016/j.jval.2015.11.007>

50. Hanney S, Davies A, Buxton M: **Assessing benefits from health research projects: can we use questionnaires instead of case studies?** *Research Evaluation* 1999, **8**(3):189-199.

51. Hanney S, Packwood T, Buxton M: **Evaluating the Benefits from Health Research and Development Centres A Categorization, a Model and Examples of Application**. *Evaluation* 2000, **6**(2):137-160.

52. Hanney S, Mugford M, Grant J, Buxton M: **Assessing the benefits of health research: lessons from research into the use of antenatal corticosteroids for the prevention of neonatal respiratory distress syndrome**. *Social science & medicine (1982)* 2005, **60**(5):937-947. <https://dx.doi.org/10.1016/j.socscimed.2004.06.038>

53. Hanney SR, Home PD, Frame I, Grant J, Green P, Buxton MJ: **Identifying the impact of diabetes research**. *Diabetic Medicine* 2006, **23**(2):176-184. <http://dx.doi.org/10.1111/j.1464-5491.2005.01753.x>

54. Hanney S, Buxton M, Green C, Coulson D, Raftery J: **An assessment of the impact of the NHS Health Technology Assessment Programme**. *Health Technology Assessment* 2007, **11**(53):iii.

55. Hanney SR, Watt A, Jones TH, Metcalf L: **Conducting retrospective impact analysis to inform a medical research charity’s funding strategies: the case of Asthma UK**. *Allergy, Asthma, and Clinical Immunology : Official Journal of the Canadian Society of Allergy and Clinical Immunology* 2013, **9**(1):17-17. <https://dx.doi.org/10.1186/1710-1492-9-17>

56. Harpham T, Tuan T: **From research evidence to policy: Mental health care in Viet Nam**. *Bull World Health Organ* 2006, **84**(8):664-668. <https://www.ncbi.nlm.nih.gov/pmc/articles/PMC2627437/pdf/16917656.pdf>

57. Hegger I, Janssen SW, Keijsers JF, Schuit AJ, van Oers HA: **Analyzing the contributions of a government-commissioned research project: a case study**. *Health Res Policy Syst* 2014, **12**:8. <https://dx.doi.org/10.1186/1478-4505-12-8>

58. Hughes CE: **Evidence-based policy or policy-based evidence? The role of evidence in the development and implementation of the Illicit Drug Diversion Initiative**. *Drug Alcohol Rev* 2007, **26**(4):363-368. <https://dx.doi.org/10.1080/09595230701373859>

59. Hunsmann M: **Limits to evidence-based health policymaking: policy hurdles to structural HIV prevention in Tanzania**. *Social science & medicine (1982)* 2012, **74**(10):1477-1485. <https://dx.doi.org/10.1016/j.socscimed.2012.01.023>

60. Hutchinson E: **The development of health policy in Malawi: The influence of context, evidence and links in the creation of a national policy for cotrimoxazole prophylaxis**. *Malawi Medical Journal : The Journal of Medical Association of Malawi* 2011, **23**(4):109-114. <http://www.ncbi.nlm.nih.gov/pmc/articles/PMC3588576/>

61. Hyde J, Mackie T, Palinkas L, Niemi E, Leslie L: **Evidence Use in Mental Health Policy Making for Children in Foster Care**. *Adm Policy Ment Health* 2015:1-15. 10.1007/s10488-015-0633-1 <http://dx.doi.org/10.1007/s10488-015-0633-1>

62. Innvær S: **The use of evidence in public governmental reports on health policy: an analysis of 17 Norwegian official reports (NOU)**. *BMC Health Services Research* 2009, **9**:177-177. <https://dx.doi.org/10.1186/1472-6963-9-177>

63. Ir P, Bigdeli M, Meessen B, Van Damme W: **Translating knowledge into policy and action to promote health equity: The Health Equity Fund policy process in Cambodia 2000-2008**. *Health Policy* 2010, **96**(3):200-209. <https://dx.doi.org/10.1016j.healthpol.2010.02.003>

64. Jacob R, McGregor M: **Assessing the impact of health technology assessment**. *Int J Technol Assess Health Care* 1997, **13**(1):68-80.

65. Johns DM, Bayer R, Fairchild AL: **Evidence and the Politics of Deimplementation: The Rise and Decline of the "counseling and Testing" Paradigm for HIV Prevention at the US Centers for Disease Control and Prevention**. *Milbank Quarterly* 2016, **94**(1):126-162. <http://dx.doi.org/10.1111/1468-0009.12183>

66. Kalucy EC, Jackson-Bowers E, McIntyre E, Reed R: **The feasibility of determining the impact of primary health care research projects using the Payback Framework**. *Health Research Policy and Systems* 2009, **7**(1):11. 10.1186/1478-4505-7-11 <http://dx.doi.org/10.1186/1478-4505-7-11>

67. Kingwell B, Anderson G, Duckett S, Hoole E, Jackson-Pulver L, Khachigian L, Morris M, Roder D, Rothwell-Short J, Wilson A: **Evaluation of NHMRC funded research completed in 1992, 1997 and 2003: gains in knowledge, health and wealth**. *Medical journal of Australia* 2006, **184**(6):282-286.

68. Kite HA, Gollust SE, Callanan RA, Weisman SR, Benning SJ, Nanney MS: **Uses of Research Evidence in the State Legislative Process to Promote Active Environments in Minnesota**. *American Journal of Health Promotion* 2014, **28**(sp3):S44-S46. <https://dx.doi.org/10.4278/ajhp.130430-ARB-217>

69. Kok MO, Gyapong JO, Wolffers I, Ofori-Adjei D, Ruitenberg J: **Which health research gets used and why? An empirical analysis of 30 cases**. *Health Research Policy and Systems* 2016, **14 (1) (no pagination)**(36).

70. Kryl D, Allen L, Dolby K, Sherbon B, Viney I: **Tracking the impact of research on policy and practice: investigating the feasibility of using citations in clinical guidelines for research evaluation**. *BMJ open* 2012, **2**(2):e000897.

71. Kurko T, Silvast A, Wahlroos H, Pietila K, Airaksinen M: **Is pharmaceutical policy evidence-informed? A case of the deregulation process of nicotine replacement therapy products in Finland**. *Health Policy* 2012, **105**(2-3):246-255. <https://dx.doi.org/10.1016/j.healthpol.2012.02.013>

72. Kuruvilla S, Mays N, Walt G: **Describing the impact of health services and policy research**: Journal of Health Services Research & Policy. 12(Suppl 1):S1-23-31, 2007.

73. Kwan P, Johnston J, Fung AY, Chong DS, Collins RA, Lo SV: **A systematic evaluation of payback of publicly funded health and health services research in Hong Kong**. *BMC Health Services Research* 2007, **7 (no pagination)**(121). <http://dx.doi.org/10.1186/1472-6963-7-121>

74. Lavis JN, Ross SE, Hurley JE: **Examining the role of health services research in public policymaking**. *Milbank quarterly* 2002, **80**(1):125-154.

75. Laws R, King L, Hardy LL, Milat A, Rissel C, Newson R, Rychetnik L, Bauman AE: **Utilization of a population health survey in policy and practice: a case study**. *Health Res Policy Syst* 2013, **11**(4).

76. Lewison G, Sullivan R: **The impact of cancer research: how publications influence UK cancer clinical guidelines**. *British Journal of Cancer* 2008, **98**(12):1944-1950. <https://dx.doi.org/10.1038/sj.bjc.6604405>

77. Lewison G: **Beyond outputs: new measures of biomedical research impact**. In: *Aslib Proceedings: 2003*: MCB UP Ltd; 2003: 32-42.

78. Liebow E, Phelps J, Van Houten B, Rose S, Orians C, Cohen J, Monroe P, Drew CH: **Toward the assessment of scientific and public health impacts of the National Institute of Environmental Health Sciences Extramural Asthma Research Program using available data**. *Environ Health Perspect* 2009, **117**(7):1147-1154. <https://dx.doi.org/10.1289/ehp.0800476>

79. Linley WG, Hughes DA: **Reimbursement decisions of the All Wales Medicines Strategy Group: influence of policy and clinical and economic factors**. *Pharmacoeconomics* 2012, **30**(9):779-794. <https://dx.doi.org/10.216511591530-000000000-00000>

80. Macnaughton E, Nelson G, Goering P: **Bringing politics and evidence together: policy entrepreneurship and the conception of the At Home/Chez Soi Housing First Initiative for addressing homelessness and mental illness in Canada**. *Social science & medicine (1982)* 2013, **82**:100-107. <https://dx.doi.org/10.1016/j.socscimed.2013.01.033>

81. Mbachu CO, Onwujekwe O, Chikezie I, Ezumah N, Das M, Uzochukwu BS: **Analysing key influences over actors' use of evidence in developing policies and strategies in Nigeria: a retrospective study of the Integrated Maternal Newborn and Child Health strategy**. *Health Research Policy & Systems*, **14**:27.

82. McCarthy M, Alexanderson K, Voss M, Conceicao C, Grimaud O, Narkauskaite L, Katreniakova Z, Saliba A, Sammut M: **Impact of innovations in national public health markets in Europe**. *European journal of public health* 2013, **23 Suppl 2**:25-29. <https://dx.doi.org/10.1093/eurpub/ckt151>

83. McKee M, Fulop N, Bouvier P, Hort A, Brand H, Rasmussen F, Kohler L, Varasovszky Z, Rosdahl N: **Preventing sudden infant deaths--the slow diffusion of an idea**. *Health Policy* 1996, **37**(2):117-135.

84. Meagher L, Lyall C, Nutley S: **Flows of knowledge, expertise and influence: a method for assessing policy and practice impacts from social science research**. *Research Evaluation* 2008, **17**(3):163-173. <http://dx.doi.org/10.3152/095820208X331720>

85. Milat AJ, Laws R, King L, Newson R, Rychetnik L, Rissel C, Bauman AE, Redman S, Bennie J: **Policy and practice impacts of applied research: a case study analysis of the New South Wales Health Promotion Demonstration Research Grants Scheme 2000–2006**. *Health Res Policy Syst* 2013, **11**(1):5.

86. Milton K, Grix J: **Public health policy and walking in England-analysis of the 2008 'policy window'**. *BMC Public Health* 2015, **15**:614. <http://dx.doi.org/10.1186/s12889-015-1915-y>

87. Molas-Gallart J, Tang P, Morrow S: **Assessing the non-academic impact of grant-funded socio-economic research: results from a pilot study**. *Research Evaluation* 2000, **9**(3):171-182. 10.3152/147154400781777269 http://dx.doi.org/10.3152/147154400781777269

88. Morton S: **Creating research impact: the roles of research users in interactive research mobilisation**. *Evidence & Policy: A Journal of Research, Debate and Practice* 2015, **11**(1):35-55. <https://dx.doi.org/10.1332/174426514X13976529631798>

89. Mubyazi GM, Gonzalez-Block MA: **Research influence on antimalarial drug policy change in Tanzania: case study of replacing chloroquine with sulfadoxine-pyrimethamine as the first-line drug**. *Malar J* 2005, **4**:51. <https://dx.doi.org/10.1186/1475-2875-4-51>

90. Nabyonga-Orem J, Ssengooba F, Mijumbi R, Tashobya CK, Marchal B, Criel B: **Uptake of evidence in policy development: the case of user fees for health care in public health facilities in Uganda**. *BMC Health Services Research* 2014, **14**:639. <https://dx.doi.org/10.1186/s12913-014-0639-5>

91. Nabyonga-Orem J, Ssengooba F, Mac QJ, Criel B: **Malaria treatment policy change in Uganda: What role did evidence play?** *Malar J* 2014, **13 (1) (no pagination)**(345). <http://dx.doi.org/10.1186/1475-2875-13-345>

92. Nabyonga-Orem J, Nanyunja M, Marchal B, Criel B, Ssengooba F: **The roles and influence of actors in the uptake of evidence: the case of malaria treatment policy change in Uganda**. *Implementation Science* 2014, **9**:150. <https://dx.doi.org/10.1186s13012-014-0150-8>

93. Nason E, Curran B, Hanney S, Janta B, Hastings G, O'Driscoll M, Wooding S: **Evaluating health research funding in Ireland: assessing the impacts of the Health Research Board of Ireland's funding activities**. *Research Evaluation* 2011, **20**(3):193-200.

94. Nathan SA, Develin E, Grove N, Zwi AB: **An Australian childhood obesity summit: the role of data and evidence in 'public' policy making**. *Aust New Zealand Health Policy* 2005, **2**:17. 10.1186/1743-8462-2-17 <https://www.ncbi.nlm.nih.gov/pubmed/16029512>

95. Newson R, King L, Rychetnik L, Bauman AE, Redman S, Milat AJ, Schroeder J, Cohen G, Chapman S: **A mixed methods study of the factors that influence whether intervention research has policy and practice impacts: perceptions of Australian researchers**. *BMJ Open* 2015, **5**(7):e008153. <http://dx.doi.org/10.1136/bmjopen-2015-008153>

96. Onwujekwe O, Uguru N, Russo G, Etiaba E, Mbachu C, Mirzoev T, Uzochukwu B: **Role and use of evidence in policymaking: an analysis of case studies from the health sector in Nigeria**. *Health Res Policy Syst* 2015, **13**:46. <https://dx.doi.org/10.1186/s12961-015-0049-0>

97. Oortwijn WJ, Hanney SR, Ligtvoet A, Hoorens S, Wooding S, Grant J, Buxton MJ, Bouter LM: **Assessing the impact of health technology assessment in The Netherlands**. *Int J Technol Assess Health Care* 2008, **24**(3):259-269. <https://dx.doi.org/10.1017/s0266462308080355>

98. Orians CE, Abed J, Drew CH, Rose SW, Cohen JH, Phelps J: **Scientific and public health impacts of the NIEHS Extramural Asthma Research Program: insights from primary data**. *Research Evaluation* 2009, **18**(5):375-385.

99. Ottoson JM, Green LW, Beery WL, Senter SK, Cahill CL, Pearson DC, Greenwald HP, Hamre R, Leviton L: **Policy-contribution assessment and field-building analysis of the Robert Wood Johnson Foundation's Active Living Research Program**. *American journal of preventive medicine* 2009, **36**(2 Suppl):S34-43. <https://dx.doi.org/10.1016/j.amepre.2008.10.010>

100. Ottoson JM, Ramirez AG, Green LW, Gallion KJ: **Exploring Potential Research Contributions to Policy**. *American journal of preventive medicine* 2013, **44**(3):S282-S289. <http://dx.doi.org/10.1016/j.amepre.2012.11.025>

101. Oxman AD, Lavis JN, Fretheim A: **Use of evidence in WHO recommendations**. *The Lancet*, **369**(9576):1883-1889. <http://dx.doi.org/10.1016/S0140-6736(07)60675-8>

102. Parkhurst JO: **Framing, ideology and evidence: Uganda's HIV success and the development of PEPFAR's 'ABC' policy for HIV prevention**. *Evidence & Policy: A Journal of Research, Debate and Practice* 2012, **8**(1):17-36. <https://doi.org/10.1332/174426412X620119>

103. PausJenssen AM, Singer PA, Detsky AS: **Ontario's formulary committee: how recommendations are made**. *Pharmacoeconomics* 2003, **21**(4):285-294.

104. Pearson M, Zwi AB, Buckley NA, Manuweera G, Fernando R, Dawson AH, McDuie-Ra D: **Policymaking 'under the radar': a case study of pesticide regulation to prevent intentional poisoning in Sri Lanka**. *Health policy and planning* 2015, **30**(1):56-67. <http://dx.doi.org/10.1093/heapol/czt096>

105. Philpott A, Maher D, Grosskurth H: **Translating HIV/AIDS research findings into policy: lessons from a case study of ‘the Mwanza trial’**. *Health Policy and Planning* 2002, **17**(2):196-201. <http://dx.doi.org/10.1093/heapol/17.2.196>

106. Probandari A, Widjanarko B, Mahendradhata Y, Sanjoto H, Cerisha A, Nungky S, Riono P, Simon S, Farid MN, Giriputra S *et al*: **The path to impact of operational research on tuberculosis control policies and practices in Indonesia**. *Glob Health Action* 2016, **9**:29866. <https://dx.doi.org/10.3402/gha.v9.29866>

107. Raftery J, Hanney S, Green C, Buxton M: **Assessing the impact of England's National Health Service R&D Health Technology Assessment program using the "payback" approach**. *Int J Technol Assess Health Care* 2009, **25**(1):1-5. <https://dx.doi.org/10.1017/s0266462309090011>

108. Reed RL, Kalucy EC, Jackson-Bowers E, McIntyre E: **What research impacts do Australian primary health care researchers expect and achieve?** *Health Research Policy and Systems* 2011, **9**(1):40. 10.1186/1478-4505-9-40 <https://doi.org/10.1186/1478-4505-9-40>

109. Rispel LC, Doherty J: **Research in support of health systems transformation in South Africa: The experience of the Centre for Health Policy**. *Journal of Public Health Policy* 2011, **32**(1):S10-S29. <http://dx.doi.org/10.1057/jphp.2011.33>

110. Ritter A, Lancaster K: **Measuring research influence on drug policy: A case example of two epidemiological monitoring systems**. *Int J Drug Policy* 2013, **24**(1):30-37.

111. Rosella LC, Wilson K, Crowcroft NS, Chu A, Upshur R, Willison D, Deeks SL, Schwartz B, Tustin J, Sider D *et al*: **Pandemic H1N1 in Canada and the use of evidence in developing public health policies--a policy analysis**. *Social Science & Medicine* 2013, **83**:1-9. <https://dx.doi.org/10.1016j.socscimed.2013.02.009>

112. Sainty M: **Research impact: a United Kingdom Occupational Therapy Research Foundation perspective**. *British Journal of Occupational Therapy* 2013, **76**(12):528-537. <https://dx.doi.org/10.4276/030802213x13861576675204>

113. Savoie I, Kazanjian A, Bassett K: **Do clinical practice guidelines reflect research evidence?** *Journal of health services research & policy* 2000, **5**(2):76-82. <https://dx.doi.org/10.1177/135581960000500204>

114. Schumacher I, Zechmeister I: **Assessing the impact of health technology assessment on the austrian healthcare system**. *International Journal of Technology Assessment in Health Care* 2013, **29**(1):84-91. <https://dx.doi.org/10.1017/s0266462312000748>

115. Scott JE, Blasinsky M, Dufour M, Mandai RJ, Philogene GS: **An evaluation of the Mind-Body Interactions and Health Program: assessing the impact of an NIH program using the Payback Framework**. *Research Evaluation* 2011, **20**(3):185-192.

116. Shah S, Ward JE: **Outcomes from NHMRC public health research project grants awarded in 1993**. *Australian and New Zealand journal of public health* 2001, **25**(6):556-560.

117. Sharma T, Choudhury M, Kaur B, Naidoo B, Garner S, Littlejohns P, Staniszewska S: **Evidence informed decision making: the use of “colloquial evidence” at nice**. *International Journal of Technology Assessment in Health Care* 2015, **31**(03):138-146. <http://dx.doi.org/10.1017/S0266462314000749>

118. Shearer JC, Dion M, Lavis JN: **Exchanging and using research evidence in health policy networks: a statistical network analysis**. *Implement Sci* 2014, **9**:126. <https://dx.doi.org/10.1186/s13012-014-0126-8>

119. Snooks HA, Kingston MR, Anthony RE, Russell IT: **New Models of Emergency Prehospital Care That Avoid Unnecessary Conveyance to Emergency Department: Translation of Research Evidence into Practice?** *The Scientific World Journal* 2013, **2013**:6. <http://dx.doi.org/10.1155/2013/182102>

120. Solans-Domenech M, Adam P, Guillamon I, Permanyer-Miralda G, Pons JMV, Escarrabill J: **Impact of clinical and health services research projects on decision-making: A qualitative study**. *Health Research Policy and Systems* 2013, **11**(1). <http://dx.doi.org/10.1186/1478-4505-11-15>

121. Soper B, Hanney SR: **Lessons from the evaluation of the UK's NHS R&D Implementation Methods Programme**. *Implementation Science* 2007, **2**:7-7. <https://dx.doi.org/10.1186/1748-5908-2-7>

122. Strehlenert H, Richter-Sundberg L, Nystrom ME, Hasson H: **Evidence-informed policy formulation and implementation: a comparative case study of two national policies for improving health and social care in Sweden**. *Implementation Science* 2015, **10**:169. <https://dx.doi.org/10.1186/s13012-015-0359-1>

123. Stryer D, Tunis S, Hubbard H, Clancy C: **The outcomes of outcomes and effectiveness research: impacts and lessons from the first decade**. *Health Services Research* 2000, **35**(5 Pt 1):977-993. <http://www.ncbi.nlm.nih.gov/pmc/articles/PMC1089179/>

124. Theobald S, Taegtmeyer M, Squire SB, Crichton J, Simwaka BN, Thomson R, Makwiza I, Tolhurst R, Martineau T, Bates I: **Towards building equitable health systems in Sub-Saharan Africa: lessons from case studies on operational research**. *Health Research Policy and Systems* 2009, **7**(1):26. <https://doi.org/10.1186/1478-4505-7-26>

125. Tomson G, Paphassarang C, Jonsson K, Houamboun K, Akkhavong K, Wahlstrom R: **Decision-makers and the usefulness of research evidence in policy implementation--a case study from Lao PDR**. *Social science & medicine (1982)* 2005, **61**(6):1291-1299. <https://dx.doi.org/10.1016/j.socscimed.2005.01.014>

126. Toner P, Lloyd C, Thom B, MacGregor S, Godfrey C, Herring R, Tchilingirian J: **Perceptions on the role of evidence: an English alcohol policy case study**. *Evidence & Policy: A Journal of Research, Debate and Practice* 2014, **10**(1):93-112. <https://dx.doi.org/10.1332/10.1332/174426514x13899745453819>

127. Tran NT, Bennett SC, Bishnu R, Singh S: **Analyzing the sources and nature of influence: how the Avahan program used evidence to influence HIV/AIDS prevention policy in India**. *Implementation Science* 2013, **8**:44. <https://dx.doi.org/10.1186/1748-5908-8-44>

128. Trostle J, Bronfman M, Langer A: **How Do Researchers Influence Decision-Makers? Case Studies of Mexican Policies**. *Health Policy and Planning* 1999, **14**(2):103-114. 10.1093/heapol/14.2.103 <http://dx.doi.org/10.1093/heapol/14.2.103>

129. Tulloch O, Mayaud P, Adu-Sarkodie Y, Opoku BK, Lithur NO, Sickle E, Delany-Moretlwe S, Wambura M, Changalucha J, Theobald S: **Using research to influence sexual and reproductive health practice and implementation in Sub-Saharan Africa: A case-study analysis**. *Health Research Policy and Systems* 2011, **9**(SUPPL. 1). <http://dx.doi.org/10.1186/1478-4505-9-S1-S10>

130. Utens CM, van der Weijden T, Joore MA, Dirksen CD: **The use of research evidence on patient preferences in pharmaceutical coverage decisions and clinical practice guideline development: exploratory study into current state of play and potential barriers**. *BMC Health Services Research* 2014, **14**:540. <https://dx.doi.org/10.1186s12913-014-0540-2>

131. van den Heuvel WJA, Wieringh R, van den Heuvel LPM: **Utilisation of medical technology assessment in health policy**. *Health Policy* 1997, **42**(3):211-222. <http://dx.doi.org/10.1016/S0168-8510(97)00073-0>

132. Voss M, Alexanderson K, McCarthy M: **Tracking uptake of innovations from the European Union Public Health Programme**. *European journal of public health* 2013, **23 Suppl 2**:19-24. <https://dx.doi.org/10.1093/eurpub/ckt150>

133. Walugembe DR, Kiwanuka SN, Matovu JKB, Rutebemberwa E, Reichenbach L: **Utilization of research findings for health policy making and practice: Evidence from three case studies in Bangladesh**. *Health Research Policy and Systems* 2015, **13 (1) (no pagination)**(26). <http://dx.doi.org/10.1186/s12961-015-0015-x>

134. Warner KE, Tam J: **The impact of tobacco control research on policy: 20 years of progress**. *Tobacco Control* 2012, **21**(2):103-109. <https://dx.doi.org/10.1136/tobaccocontrol-2011-050396>

135. Weatherly H, Drummond M, Smith D: **Using evidence in the development of local health policies. Some evidence from the United Kingdom**. *Int J Technol Assess Health Care* 2002, **18**(4):771-781.

136. Whiteside A, Henry FE: **The impact of HIV and AIDS research: a case study from Swaziland**. *Health Research Policy and Systems* 2011, **9**(1):S9. 10.1186/1478-4505-9-s1-s9 <http://dx.doi.org/10.1186/1478-4505-9-S1-S9>

137. Williams I, McIver S, Moore D, Bryan S: **The use of economic evaluations in NHS decision-making: a review and empirical investigation**. *Health Technology Assessment* 2008, **12**(7):196. <https://dx.doi.org/10.3310/hta12070>

138. Wilson PM, Petticrew M, Calnan MW, Nazareth I: **Does dissemination extend beyond publication: a survey of a cross section of public funded research in the UK**. *Implementation Science* 2010, **5**(1):61. 10.1186/1748-5908-5-61 <http://dx.doi.org/10.1186/1748-5908-5-61>

139. Woelk G, Daniels K, Cliff J, Lewin S, Sevene E, Fernandes B, Mariano A, Matinhure S, Oxman AD, Lavis JN *et al*: **Translating research into policy: lessons learned from eclampsia treatment and malaria control in three southern African countries**. *Health Res Policy Syst* 2009, **7**:31. <https://dx.doi.org/10.1186/1478-4505-7-31>

140. Wooding S, Hanney SR, Pollitt A, Grant J, Buxton MJ, Project Retrosight T: **Understanding factors associated with the translation of cardiovascular research: a multinational case study approach**. *Implementation Science* 2014, **9**(1):47. <https://dx.doi.org/10.1186/1748-5908-9-47>

141. Wooding S, Hanney S, Buxton M, Grant J: **Payback arising from research funding: evaluation of the Arthritis Research Campaign**. *Rheumatology* 2005, **44**(9):1145-1156. <http://dx.doi.org/10.1093/rheumatology/keh708>

142. Yazdizadeh B, Majdzadeh R, Janani L, Mohtasham F, Nikooee S, Mousavi A, Najafi F, Atabakzadeh M, Bazrafshan A, Zare M *et al*: **An assessment of health research impact in Iran**. *Health Research Policy and Systems* 2016, **14 (1)** (56).

143. Zardo P, Collie A: **Measuring use of research evidence in public health policy: a policy content analysis**. *BMC Public Health* 2014, **14**:496. <https://dx.doi.org/10.1186/1471-2458-14-496>

144. Zechmeister I, Schumacher I: **The impact of health technology assessment reports on decision making in austria**. *International Journal of Technology Assessment in Health Care* 2012, **28**(1):77-84. <https://dx.doi.org/10.1017/s0266462311000729>
